# NLR-parser: rapid annotation of plant NLR complements

**DOI:** 10.1093/bioinformatics/btv005

**Published:** 2015-01-12

**Authors:** Burkhard Steuernagel, Florian Jupe, Kamil Witek, Jonathan D.G. Jones, Brande B.H. Wulff

**Affiliations:** ^1^Department of Crop Genetics, John Innes Centre, Norwich, UK and ^2^The Sainsbury Laboratory, Norwich, UK

## Abstract

**Motivation:** The repetitive nature of plant disease resistance genes encoding for nucleotide-binding leucine-rich repeat (NLR) proteins hampers their prediction with standard gene annotation software. Motif alignment and search tool (MAST) has previously been reported as a tool to support annotation of NLR-encoding genes. However, the decision if a motif combination represents an NLR protein was entirely manual.

**Results:** The NLR-parser pipeline is designed to use the MAST output from six-frame translated amino acid sequences and filters for predefined biologically curated motif compositions. Input reads can be derived from, for example, raw long-read sequencing data or contigs and scaffolds coming from plant genome projects. The output is a tab-separated file with information on start and frame of the first NLR specific motif, whether the identified sequence is a TNL or CNL, potentially full or fragmented. In addition, the output of the NB-ARC domain sequence can directly be used for phylogenetic analyses. In comparison to other prediction software, the highly complex NB-ARC domain is described in detail using several individual motifs.

**Availability and implementation:** The NLR-parser tool can be downloaded from Git-Hub (github.com/steuernb/NLR-Parser). It requires a valid Java installation as well as MAST as part of the MEME Suite. The tool is run from the command line.

**Contact:** burkhard.steuernagel@jic.ac.uk; fjupe@salk.edu

**Supplementary information:** Supplementary data are available at *Bioinformatics* online.

## 1 Introduction

Plants have evolved a multi-layered innate immune system to protect themselves against pests and pathogens (Jones and Dangl, 2006). Breeding efforts towards disease resistance in crops rely on the introgression of quantitative trait loci or major dominant disease resistance (*R*) genes from wild relatives (reviewed in (Dangl *et al*., 2013). The largest class of *R* genes encodes nucleotide-binding domain leucine-rich repeat proteins (NLRs or NB-LRRs). These are key receptors that recognize secreted pathogen effector molecules or their effect in the plant. On recognition, these proteins commonly lead to a hypersensitive response in the form of local cell death to prevent further spread of pathogens relying on living tissue (Jones and Dangl, 2006).

In dicotyledonous plants, NLR proteins come in two flavours that are determined by an N-terminal extension and internal amino acid motif composition. CNL proteins possess in most cases a coiled-coil domain followed by the highly conserved p-loop and RNBS-A motif (Meyers *et al.*, 2003). TNL proteins possess a Toll-interleukin receptor-like (TIR) domain followed by the p-loop but lack the RNBS-A motif. The TNL class is absent from monocotyledonous plants, like wheat and barley. A set of 20 NLR descriptive motifs have previously been identified using MEME (Bailey *et al.*, 2009), and were used in motif alignment and search tool (MAST) searches against predicted potato proteins (Jupe *et al*., 2012). Originally set out to discover NLR sequences from members of the plant family Solanaceae, this set also contains two Triticeae specific motifs.

The identification and annotation of the very large NLR gene family, with for example over 750 members in potato, is currently very laborious and time-consuming, as most automated gene callers fail to capture the full complement. Several studies have shown that these automated annotations miss up to 50% of the total NLR gene complement, or that full sequences are split into small fragments and then annotated as ‘partial’ (Meyers *et al.*, 2003; Jupe *et al*., 2013; Andolfo *et al*., 2014).

There is, therefore, a clear need for an automated NLR annotation tool. Here, we present an NLR-MAST-parser, a java application for the identification of NLR-like sequences that uses the highly specific amino acid motif composition found in plant NLR gene products and parses this information into an easy-to-use tabular file. The impact of this tool comes from a high accuracy, reduction in hands-on time of NLR annotation projects and its independence from gene prediction software. We further provide evidence that it is functional in monocotyledonous and dicotyledonous plant species.

## 2 Methods

### 2.1 Motif composition discriminates NLRs

The amino acid motif composition of NLR gene products is highly conserved amongst all plant species, sufficient to separate these from other protein sequences and sufficient to separate the two main types of NLRs (TNL and CNL). We use 20 previously biologically characterized motifs (Jupe *et al.*, 2012) in the MAST tool to identify potential NLR encoding sequences. The NLR parser uses a variety of biologically defined input motif compositions to search the MAST xml-format output and report on confirmed NLRs only. These motif compositions can be found in the online manual.

### 2.2 Mast parser features

The annotation of NLR genes is a manual process that is simplified by several output features of this NLR parser. The MAST input is a protein sequence, which is usually not available from, for example *de novo* assembled genomes or NLR-enriched sequence data. The best procedure to identify NLRs in a set of sequences is to perform a translation into all six reading frames. The MAST Parser accepts a pattern, which splits a common prefix from frame-specific suffixes, as an input argument. That way, every nucleotide sequence can be annotated, regardless of the actual reading frame or even a shift of the frame. It has been shown that NLR genes are often under selection (Michelmore and Meyers, 1998), resulting in a large number of pseudogenes. We defined sets of motifs that indicate the completeness of an NLR gene. The output of the Mast Parser includes this annotation as a column. Finally, we add the class of each NLR, i.e. CNL or TNL, to the output.

### 2.3 TAIR validation

In a proof-of-concept study, we screened the available set of *Arabidopsis thaliana* TAIR proteins (TAIR10_pep_20101214) for NLR gene products using the here presented MAST pipeline. In total, we identified 266 from within 35 386 Arabidopsis proteins as partial or complete NLRs. The original TAIR protein annotation provides 219 sequences with one of the following annotation terms: ‘Toll-Interleukin-Resistance (TIR) domain’, ‘NB-ARC’ or ‘NBS-LRR’ and 212 of these were also identified with our MAST pipeline. Blastp analyses of the seven remaining proteins identified two false-negatives with an NB-ARC and LRR domains, but five that had neither an NB-ARC nor an LRR domain and thus can be excluded. Detailed analysis shows that the two false-negatives correspond to the ancient and small group of NLRs with similarity to *ADR1* (Chini and Loake, 2005). Here, the discriminatory Motif 8 had a *P*-value of 8e−5 and was, therefore, discarded. We, therefore, observe a sensitivity of more than 99%. We found five complete NLRs with the NLR-Parser that were not annotated accordingly in TAIR. We validated the structure of those proteins by scanning for TIR, NB-ARC and LRR-related PFAM domains using HMMER (Eddy, 2011) and found consistently an NB-ARC domain and LRRs in each of the protein sequences (Supplementary Table 1). Therefore, we report a 100% specificity for the NLR-Parser.

### 2.4 Monocot validation

We further tested the MEME motifs in our NLR-parser for their functionality in monocotyledonous plant genomes and screened the publicly available set of annotated genes from *Brachypodium distachyon*. The NLR-parser pipeline identified 586 partial or complete NLRs. All 190 proteins that the NLR-parser annotated as complete NLRs have previously been annotated as resistance genes (http://phytozome.jgi.doe.gov/). The general quality of the *Brachypodium* annotation, relying on similarity to Arabidopsis and rice does not allow a precise estimation of sensitivity and selectivity. However, there is a good consistency between annotation, found PFAM domains and NLR-Parser. Eight genes with NB-ARC domain and LRR have not been found by the NLR-Parser, including an *ADR1-like*. Conversely, the NLR-Parser annotated 47 proteins as complete NLRs while HMMER only detected the NB-ARC domain, not any LRR (Supplementary Table 2).

## 3 Discussion

Due to the biological importance and relevance for breeding, the identification and annotation of NLR-type disease resistance genes has high priority in all plant genome sequencing projects. These annotations, however, rely heavily on gene-prediction software. In the past, we were able to show that up to 50% of the total NLR complement was either wrongly predicted or completely missing. Our MAST Parser tool provides high precision identification of NLR gene sequences from every input format that is available from genome sequencing projects including contigs, scaffolds, pseudomolecules or chromosomes. In two experiments with the model plants *A. thaliana* and *B. distachyon*, we were able to show the functionality of the 20 well-characterized MEME motifs in monocotyledonous and dicotyledonous plants. The output of this tool is directly usable for downstream applications including phylogenetic analyses, or visualization on the corresponding reference sequence. The tab delimited output format is publishable as a Supplementary Table.

## 4 Conclusion

The MAST Parser pipeline that we present here will streamline NLR identification efforts within genome sequencing projects in monocotyledonous and dicotyledonous plants.

## Supplementary Material

Supplementary Data
