# Supplementary material for: NLR-parser: rapid annotation of plant NLR complements
Source: Bioinformatics. 2015 Jan 12;31(10):1665–7. doi: 10.1093/bioinformatics/btv005 (PMC4426836; doi:10.1093/bioinformatics/btv005)
Supplement: Supplementary Data [file supp_31_10_1665__index.html]

NLR-parser: Rapid annotation of plant NLR complements — NLR-parser: rapid annotation of plant NLR complements — NLR-parser: rapid annotation of plant NLR complements — Supplementary Data 

# NLR-parser: rapid annotation of plant NLR complements

## Supplementary Data

files

**Files in this Data Supplement:**

- Supplementary Data - pdf file
