# Supplementary material for: NLR-parser: rapid annotation of plant NLR complements
Source: Bioinformatics. 2015 Jan 12;31(10):1665–7. doi: 10.1093/bioinformatics/btv005 (PMC4426836; doi:10.1093/bioinformatics/btv005)
Supplement: Supplementary Data [file supp_btv005_SupplementaryTables.pdf]

Supplementary Table 1: NLR annotation of *Arabidopsis thaliana* proteins using PFAM domains, NLR-Parser and text search

Gene ID	HMMER Scan	NLR Parser	TAIR Annotation
AT1G17600.1	TIR,NB-ARC,LRR	TNL;complete;18,15,13,1,4,5,10,3,12,7,11,11,9,9,11,11,9,11	Disease resistance protein (TIR-NBS-LRR class) family
AT1G10920.1	NB-ARC	CNL;partial;1,6,4,5,10,3,12,2,8,7,9,9	NB-ARC domain-containing disease resistance protein
AT1G65850.1	TIR,NB-ARC,LRR	TNL;complete;18,15,13,1,4,5,10,3,12,7,11,11,11,11,9,11,11	Disease resistance protein (TIR-NBS-LRR class) family
AT1G09665.1	TIR	TNL;partial;18,15,13	Toll-Interleukin-Resistance (TIR) domain family protein
AT1G63730.1	TIR,NB-ARC,LRR	TNL;complete;18,15,13,1,4,5,10,3,12,7,11,11,11,11,9,11,11	Disease resistance protein (TIR-NBS-LRR class) family
AT1G63750.1	NB-ARC,LRR	TNL;partial;1,4,5,10,3,7,9,11,11,11,11,11,11,9,11	Disease resistance protein (TIR-NBS-LRR class) family
AT1G63740.1	TIR,NB-ARC,LRR	TNL;partial;15,13,1,4,5,10,3,12,7,11,11,11,11,11,11	Disease resistance protein (TIR-NBS-LRR class) family
AT1G59780.1	NB-ARC	CNL;complete;17,16,1,6,4,5,10,3,12,2,8,7,9,11,11	NB-ARC domain-containing disease resistance protein
AT1G53780.1	Not Found	N/A;partial;13,1	peptidyl-prolyl cis-trans isomerases;hydrolases;nucleoside-triphosphatases;ATP binding;nucleotide binding;ATPases
AT1G56520.1	TIR,NB-ARC,LRR	TNL;complete;18,15,13,1,4,5,10,3,8,7,11,11,11,11,9,9,11	Disease resistance protein (TIR-NBS-LRR class) family
AT1G56510.1	TIR,NB-ARC,LRR	TNL;complete;18,15,13,1,4,5,10,3,7,11,11,11,11,11	Disease resistance protein (TIR-NBS-LRR class)
AT1G56540.1	TIR,NB-ARC,LRR	TNL;complete;18,15,13,1,4,5,10,3,7,11,11,11,11,9,11,11	Disease resistance protein (TIR-NBS-LRR class) family
AT1G59620.1	NB-ARC	CNL;complete;17,16,1,6,4,5,10,3,12,2,8,7,9,11	Disease resistance protein (CC-NBS-LRR class) family
AT1G53350.1	NB-ARC	CNL;complete;17,16,1,6,4,5,10,3,12,2,8,7,9,11,11	Disease resistance protein (CC-NBS-LRR class) family
AT1G50180.1	NB-ARC	CNL;complete;17,16,1,6,4,5,10,3,12,2,8,7,9,11	NB-ARC domain-containing disease resistance protein
AT1G17610.1	NB-ARC	TNL;partial;15,13,1,4,5,10,3	Disease resistance protein (TIR-NBS class)
AT1G17615.1	TIR,NB-ARC	TNL;partial;18,15,13,1,4,5	Disease resistance protein (TIR-NBS class)
AT1G27170.1	TIR,NB-ARC,LRR	TNL;complete;18,15,13,1,4,5,10,3,7,11,11,11,11,9,9,9,11,11,9,11,9,11,9,9,11,11,11,11	transmembrane receptors;ATP binding
AT1G27180.1	TIR,NB-ARC,LRR	TNL;complete;13,18,15,13,1,4,5,3,7,11,11,11,11,9,11,9,11,9,9,11,9,11,9,9,11,11,11,11	disease resistance protein (TIR-NBS-LRR class), putative
AT1G63350.1	NB-ARC,LRR	CNL;partial;17,1,6,4,5,10,3,12,2,8,7,11,9,11,11,11,11	Disease resistance protein (CC-NBS-LRR class) family
AT1G63360.1	NB-ARC,LRR	CNL;partial;17,1,6,4,5,10,3,12,2,8,7,11,9,11,9,11,11,11,11	Disease resistance protein (CC-NBS-LRR class) family
AT1G58848.1	NB-ARC,LRR	CNL;complete;17,16,1,6,4,5,10,3,12,2,8,7,9,9,11,2,11,11,11	Disease resistance protein (CC-NBS-LRR class) family
AT1G58807.1	NB-ARC,LRR	CNL;complete;17,16,1,6,4,5,10,3,12,2,8,7,9,9,9,11,11,11	Disease resistance protein (CC-NBS-LRR class) family
AT1G66090.1	TIR,NB-ARC	TNL;partial;18,15,13,1,4,5,3	Disease resistance protein (TIR-NBS class)
AT1G57630.1	TIR	TNL;partial;18,15,13	Toll-Interleukin-Resistance (TIR) domain family protein
AT1G59124.1	NB-ARC,LRR	CNL;partial;17,16,1,6,4,5,10,3,12,2,8,7,9,9,9	Disease resistance protein (CC-NBS-LRR class) family
AT1G57670.1	TIR	TNL;partial;18,15,13,17	Toll-Interleukin-Resistance (TIR) domain family protein
AT1G57650.1	NB-ARC,LRR	N/A;partial;10,3,12,7,11,11,11,11	ATP binding
AT1G59218.1	NB-ARC,LRR	CNL;complete;17,16,1,6,4,5,10,3,12,2,8,7,9,9,11,2,11,11,11	Disease resistance protein (CC-NBS-LRR class) family
AT1G61180.1	NB-ARC,LRR	CNL;partial;1,6,4,5,10,3,12,2,7,11,9,11,11,11,11	LRR and NB-ARC domains-containing disease resistance protein
AT1G61190.1	NB-ARC,LRR	CNL;partial;1,6,4,5,10,3,12,2,7,11,9,11,11,11,11	LRR and NB-ARC domains-containing disease resistance protein
AT1G61105.1	TIR	N/A;partial;18,15	Toll-Interleukin-Resistance (TIR) domain family protein
AT1G61100.1	Not Found	Not Found	disease resistance protein (TIR class), putative
AT1G61300.1	NB-ARC,LRR	CNL;partial;1,6,4,5,10,3,12,2,7,11,9,11,11,11,11	LRR and NB-ARC domains-containing disease resistance protein
AT1G61310.1	NB-ARC,LRR	CNL;partial;1,6,4,5,10,3,12,2,7,11,9,11,11,11,11	LRR and NB-ARC domains-containing disease resistance protein
AT1G57850.1	TIR	TNL;partial;18,15,13	Toll-Interleukin-Resistance (TIR) domain family protein
AT1G51480.1	NB-ARC,LRR	CNL;partial;1,6,4,5,10,3,12,2,7,11,9,11,11,11,11	Disease resistance protein (CC-NBS-LRR class) family
AT1G31540.1	TIR,NB-ARC,LRR	TNL;complete;18,15,13,1,4,5,10,3,12,7,11,11,11,11,9	Disease resistance protein (TIR-NBS-LRR class) family
AT1G65390.1	TIR	TNL;partial;18,15,13	phloem protein 2 A5
AT1G57830.1	TIR	TNL;partial;18,15,13	Toll-Interleukin-Resistance (TIR) domain family protein
AT1G69550.1	TIR,NB-ARC,LRR	TNL;complete;18,15,13,1,4,5,10,3,12,7,11,11,11,11,11,11,11,11,11,11,11,11,11,11,11,11,11,11,11,11,11,11	disease resistance protein (TIR-NBS-LRR class)
AT1G47370.1	TIR	TNL;partial;18,15,13	Toll-Interleukin-Resistance (TIR) domain family protein
AT1G08590.1	LRR	N/A;partial;11,9,11,11,11,11,11,9,11,11	Leucine-rich receptor-like protein kinase family protein
AT1G45616.1	LRR	N/A;partial;9,11,15,12,9,11,11,11,11	receptor like protein 6
AT1G72950.1	TIR,NB-ARC	TNL;partial;18,15,13,1,4,5,10	Disease resistance protein (TIR-NBS class)
AT1G52900.1	TIR	N/A;partial;18,15	Toll-Interleukin-Resistance (TIR) domain family protein
AT1G72850.1	TIR,NB-ARC	TNL;partial;18,15,13,1,4,5,10,3	Disease resistance protein (TIR-NBS class)
AT1G72860.1	TIR,NB-ARC,LRR	TNL;complete;18,15,13,1,4,5,10,3,12,7,11,11,11,9,11,11,9,11	Disease resistance protein (TIR-NBS-LRR class) family
AT1G72870.1	TIR,NB-ARC	TNL;partial;18,15,13,1,5,10,3	Disease resistance protein (TIR-NBS class)
AT1G72890.1	TIR,NB-ARC	TNL;partial;18,15,13,1,4,5,10	Disease resistance protein (TIR-NBS class)
AT1G52660.1	NB-ARC	CNL;partial;1,6,4,5,10,3	P-loop containing nucleoside triphosphate hydrolases superfamily protein
AT1G72900.1	TIR,NB-ARC	TNL;partial;18,15,13,1,4,5	Toll-Interleukin-Resistance (TIR) domain-containing protein
AT1G72940.1	TIR,NB-ARC	TNL;partial;18,15,13,1,4,5,10	Toll-Interleukin-Resistance (TIR) domain-containing protein
AT1G72920.1	TIR	TNL;partial;18,15,13,1	Toll-Interleukin-Resistance (TIR) domain family protein
AT1G72910.1	TIR,NB-ARC	TNL;partial;18,15,13,1,4,5	Toll-Interleukin-Resistance (TIR) domain-containing protein
AT1G72930.1	TIR	TNL;partial;18,15,13	toll/interleukin-1 receptor-like
AT1G63880.1	TIR,NB-ARC,LRR	TNL;complete;18,15,13,1,4,5,10,3,7,11,11,11,11,9,11	Disease resistance protein (TIR-NBS-LRR class) family
AT1G63870.1	TIR,NB-ARC,LRR	TNL;complete;18,15,13,1,4,5,10,3,7,9,11,11,11,11,9,11,9,11	Disease resistance protein (TIR-NBS-LRR class) family
AT1G63860.1	TIR,NB-ARC,LRR	TNL;complete;18,15,13,1,4,5,3,12,7,11,11,11,11,9,9,11	Disease resistance protein (TIR-NBS-LRR class) family
AT1G64070.1	TIR,NB-ARC,LRR	TNL;complete;18,15,13,1,4,5,10,3,11,11,11,9,9,11,11,18	Disease resistance protein (TIR-NBS-LRR class) family
AT1G33560.1	NB-ARC,LRR	Not Found	Disease resistance protein (CC-NBS-LRR class) family
AT1G72840.1	TIR,NB-ARC,LRR	TNL;complete;18,15,13,1,4,5,10,3,12,11,11,9,9,11,11,9,11,11	Disease resistance protein (TIR-NBS-LRR class)
AT1G58400.1	NB-ARC	CNL;complete;17,16,1,6,4,10,3,12,2,8,7,9,9,11,11	Disease resistance protein (CC-NBS-LRR class) family
AT1G58410.1	NB-ARC,LRR	CNL;partial;17,16,1,6,4,5,10,3,12,2,8,7,9,9,9	Disease resistance protein (CC-NBS-LRR class) family
AT1G58390.1	NB-ARC	CNL;complete;17,16,1,6,4,5,10,3,12,2,8,7,9,9,11	Disease resistance protein (CC-NBS-LRR class) family
AT1G58602.1	NB-ARC,LRR	CNL;complete;17,16,1,6,4,5,10,3,12,2,8,7,9,9,11,11,11,11,11	LRR and NB-ARC domains-containing disease resistance protein
AT1G60320.1	TIR	TNL;partial;18,15,13	Toll-Interleukin-Resistance (TIR) domain family protein
AT1G15890.1	NB-ARC,LRR	CNL;partial;1,6,4,5,10,3,12,2,7,11,9,11,11,11	Disease resistance protein (CC-NBS-LRR class) family
AT1G12280.1	NB-ARC,LRR	CNL;partial;1,6,4,5,10,3,12,2,7,11,9,11,11,11,11	LRR and NB-ARC domains-containing disease resistance protein
AT1G12210.1	NB-ARC,LRR	CNL;partial;1,6,4,5,10,3,12,2,8,7,11,11,9,11,11,11,11	RPS5-like 1
AT1G12220.1	NB-ARC,LRR	CNL;partial;17,1,6,4,5,10,3,12,2,7,11,9,11,11,11	Disease resistance protein (CC-NBS-LRR class) family
AT1G12290.1	NB-ARC,LRR	CNL;partial;17,1,6,4,5,10,3,12,2,7,11,11,9,11,11,11	Disease resistance protein (CC-NBS-LRR class) family
AT1G71390.1	LRR	N/A;partial;11,11,11,11,11,9,11,11,11,11	receptor like protein 11
AT1G04210.1	LRR	N/A;partial;9,9,11	Leucine-rich repeat protein kinase family protein
AT1G62630.1	NB-ARC,LRR	CNL;partial;17,1,6,4,5,10,3,12,2,8,7,11,9,11,11,11,11	Disease resistance protein (CC-NBS-LRR class) family
AT1G65390.2	TIR	TNL;partial;18,15,13	phloem protein 2 A5
AT1G51270.2	TIR	TNL;partial;18,15,13	structural molecules;transmembrane receptors;structural molecules
AT1G58848.2	NB-ARC,LRR	CNL;complete;17,16,1,6,4,5,10,3,12,2,8,7,9,9,11,2,11,11,11	Disease resistance protein (CC-NBS-LRR class) family
AT1G61180.2	NB-ARC,LRR	CNL;partial;1,6,4,5,10,3,12,2,7,11,9,11,11,11,11	LRR and NB-ARC domains-containing disease resistance protein
AT1G51270.1	TIR	TNL;partial;18,15,13	structural molecules;transmembrane receptors;structural molecules
AT1G58602.2	NB-ARC,LRR	CNL;complete;17,16,1,6,4,5,10,3,12,2,8,7,9,9,11,11,11,11,11	LRR and NB-ARC domains-containing disease resistance protein
AT1G63750.2	NB-ARC,LRR	TNL;partial;1,4,5,10,3,7,9,11,11,11,11,11,11,9,11	Disease resistance protein (TIR-NBS-LRR class) family
AT1G63750.3	TIR,NB-ARC,LRR	TNL;complete;18,15,13,1,4,5,10,3,7,9,11,11,11,11,11,11,9,11	Disease resistance protein (TIR-NBS-LRR class) family
AT1G72930.2	TIR	TNL;partial;18,15,13	toll/interleukin-1 receptor-like
AT1G31540.2	TIR,NB-ARC,LRR	TNL;complete;18,15,13,1,4,5,10,3,12,7,11,11,11,11,9,11,11,11,9	Disease resistance protein (TIR-NBS-LRR class) family
AT1G58807.2	NB-ARC,LRR	CNL;partial;17,16,1,6,4,5,10,3,12,2,8,7,9,9,9	Disease resistance protein (CC-NBS-LRR class) family
AT1G10920.2	NB-ARC	CNL;partial;1,6,4,5,10,3,12,2,8,7,9,9	NB-ARC domain-containing disease resistance protein
AT1G12290.2	NB-ARC,LRR	CNL;partial;1,6,4,5,10,3,12,2,7,11,11,9,11,11,11	Disease resistance protein (CC-NBS-LRR class) family
AT1G27170.2	TIR,NB-ARC,LRR	TNL;partial;15,13,1,4,5,10,3,7,11,11,11,11,9,9,9,11,11,9,11,9,11,9,9,11,11,11,11	transmembrane receptors;ATP binding
AT1G57850.2	TIR	N/A;partial;15,13	Toll-Interleukin-Resistance (TIR) domain family protein
AT1G63860.2	TIR,NB-ARC,LRR	TNL;complete;18,15,13,1,4,5,3,12,7,11,11,11,11,9,9,11	Disease resistance protein (TIR-NBS-LRR class) family

AT1G51270.3	TIR	TNL;partial;18,15,13	structural molecules;transmembrane receptors;structural molecules
AT1G65850.2	TIR,NB-ARC,LRR	TNL;complete;18,15,13,1,4,5,10,3,12,7,11,11,11,11,9,11,11	Disease resistance protein (TIR-NBS-LRR class) family
AT1G12220.2	NB-ARC,LRR	CNL;partial;17,1,6,4,5,10,3,12,2,7,11,9,11,11,11	Disease resistance protein (CC-NBS-LRR class) family
AT1G72890.2	TIR,NB-ARC	TNL;partial;18,15,13,1,4,5,10	Disease resistance protein (TIR-NBS class)
AT1G72840.2	TIR,NB-ARC,LRR	TNL;complete;18,15,13,1,4,5,10,3,12,11,11,9,9,11,11,9,11,11	Disease resistance protein (TIR-NBS-LRR class)
AT1G53780.3	Not Found	N/A;partial;13,1	peptidyl-prolyl cis-trans isomerases;hydrolases;nucleoside-triphosphatases;ATP binding;nucleotide binding;ATPases
AT1G61100.2	Not Found	Not Found	disease resistance protein (TIR class), putative
AT1G56520.2	TIR,NB-ARC,LRR	TNL;complete;18,15,13,1,4,5,10,3,8,7,11,11,11,11,9,9,11	Disease resistance protein (TIR-NBS-LRR class) family
AT1G59218.2	NB-ARC,LRR	CNL;complete;17,16,1,6,4,5,10,3,12,2,8,7,9,9,11,2,11,11,11	Disease resistance protein (CC-NBS-LRR class) family
AT1G51270.4	TIR	TNL;partial;18,15,13	structural molecules;transmembrane receptors;structural molecules
AT1G57650.2	NB-ARC,LRR	N/A;partial;10,3,12,7,11,11,11,11	ATP binding
AT2G03300.1	TIR	TNL;partial;18,15,13	Toll-Interleukin-Resistance (TIR) domain family protein
AT2G33060.1	LRR	N/A;partial;9,11,11,12,12,11,11,11	receptor like protein 27
AT2G20142.1	TIR	TNL;partial;18,15,13	Toll-Interleukin-Resistance (TIR) domain family protein
AT2G03030.1	TIR	TNL;partial;18,15,13	Toll-Interleukin-Resistance (TIR) domain family protein
AT2G32140.1	TIR	TNL;partial;18,15,13	transmembrane receptors
AT2G17050.1	TIR,NB-ARC,LRR	TNL;partial;1,8,5,10,3,12,7,11,11,9,11,11,9,11,11,9,11,11,9,11,11,18,15,13	disease resistance protein (TIR-NBS-LRR class), putative
AT2G14080.1	TIR,NB-ARC,LRR	TNL;complete;18,15,13,1,4,5,10,3,12,7,11,11,11,11,11,11,11,11,11,11,11,9,11,11	Disease resistance protein (TIR-NBS-LRR class) family
AT2G17060.1	TIR,NB-ARC,LRR	TNL;complete;18,15,13,1,4,5,3,12,7,9,11,11,11,9,11,11,9,9,11,11,19	Disease resistance protein (TIR-NBS-LRR class) family
AT2G17055.1	TIR	N/A;partial;18,15	Toll-Interleukin-Resistance (TIR) domain family protein
AT2G16870.1	TIR,NB-ARC,LRR	TNL;complete;18,15,13,1,4,5,10,3,7,9,11,11,11,11,9,11	Disease resistance protein (TIR-NBS-LRR class) family
AT2G01820.1	LRR	N/A;partial;9,11,12,11,7,11	Leucine-rich repeat protein kinase family protein
AT2G32680.1	LRR	N/A;partial;11,12,9,11,9,11,11,11,11,11,11	receptor like protein 23
AT2G34930.1	LRR	N/A;partial;11,9,11,11,11,11,11,11,11,11,11,11	disease resistance family protein / LRR family protein
AT3G15700.1	NB-ARC	CNL;partial;1,6,4,5,10,3	P-loop containing nucleoside triphosphate hydrolases superfamily protein
AT3G51570.1	TIR,NB-ARC,LRR	TNL;complete;18,15,13,1,4,5,3,12,16,7,11,11,9,11,11,9,11,11	Disease resistance protein (TIR-NBS-LRR class) family
AT3G51560.1	TIR,NB-ARC,LRR	TNL;complete;18,15,13,1,4,5,10,3,7,11,11,9,11,9,11,11,9,11,9,11,11	Disease resistance protein (TIR-NBS-LRR class) family
AT3G14470.1	NB-ARC,LRR	CNL;complete;16,1,6,4,5,10,3,12,2,8,7,9,9,11,9,19,11,11,11,11,11	NB-ARC domain-containing disease resistance protein
AT3G14460.1	NB-ARC,LRR	CNL;complete;16,1,6,4,5,10,3,12,2,8,7,9,9,11,9,19,11,11,11,11,11,11,11,11	LRR and NB-ARC domains-containing disease resistance protein
AT3G44670.1	TIR,NB-ARC,LRR	TNL;complete;18,15,13,1,4,5,10,3,12,7,11,11,11,11,11,11,11,11,11,11	Disease resistance protein (TIR-NBS-LRR class) family
AT3G44630.3	TIR,NB-ARC,LRR	TNL;complete;10,18,15,13,1,4,5,10,3,12,7,11,11,11,11,11,11,11,11,9,11	Disease resistance protein (TIR-NBS-LRR class) family
AT3G07040.1	NB-ARC,LRR	CNL;complete;17,1,16,1,6,4,5,10,3,12,2,8,7,9,9,9,11	NB-ARC domain-containing disease resistance protein
AT3G04220.1	TIR,NB-ARC,LRR	TNL;complete;18,15,13,1,4,5,10,3,12,7,11,11,11,11,11,11,11,11	Disease resistance protein (TIR-NBS-LRR class) family
AT3G44480.1	TIR,NB-ARC,LRR	TNL;complete;18,15,13,1,4,5,10,3,12,7,11,11,11,11,11,11,11,11,9,11	Disease resistance protein (TIR-NBS-LRR class) family
AT3G46710.1	NB-ARC,LRR	CNL;complete;17,16,1,6,4,5,10,3,2,8,7,9,11	NB-ARC domain-containing disease resistance protein
AT3G46730.1	NB-ARC,LRR	CNL;complete;17,16,1,6,4,5,10,3,2,8,7,9,11	NB-ARC domain-containing disease resistance protein
AT3G44400.1	TIR,NB-ARC,LRR	TNL;complete;18,15,13,1,4,5,10,3,12,7,11,11,11,11,11,11,11	Disease resistance protein (TIR-NBS-LRR class) family
AT3G25510.1	TIR,NB-ARC,LRR	TNL;complete;18,15,13,1,4,5,10,3,12,7,11,11,11,11,9,11,11,11,11,11,11,11,11,11,11,9,11,15,13,1,4,5,10,3,12,7,11,11,11,11	disease resistance protein (TIR-NBS-LRR class), putative
AT3G04210.1	TIR,NB-ARC	TNL;partial;18,15,13,1,4,5,10	Disease resistance protein (TIR-NBS class)
AT3G28890.1	LRR	N/A;partial;9,9,9,9,11,11,11,11,11,11	receptor like protein 43
AT3G11010.1	LRR	N/A;partial;9,9,9,11,11,11,9,9,11,11,11,11,11	receptor like protein 34
AT3G50950.2	NB-ARC,LRR	CNL;complete;17,16,1,6,4,5,10,3,12,2,8,7,9,11,11,11	HOPZ-ACTIVATED RESISTANCE 1
AT3G46530.1	NB-ARC	CNL;complete;17,16,1,6,4,5,10,3,2,8,7,9,11,11	NB-ARC domain-containing disease resistance protein
AT3G50950.1	NB-ARC,LRR	CNL;complete;17,16,1,6,4,5,10,3,12,2,8,7,9,11,11,11	HOPZ-ACTIVATED RESISTANCE 1
AT3G44630.2	TIR,NB-ARC,LRR	TNL;complete;10,18,15,13,1,4,5,10,3,12,7,11,11,11,11,11,11,11,11,9,11	Disease resistance protein (TIR-NBS-LRR class) family
AT3G44630.1	TIR,NB-ARC,LRR	TNL;complete;10,18,15,13,1,4,5,10,3,12,7,11,11,11,11,11,11,11,11,9,11	Disease resistance protein (TIR-NBS-LRR class) family
AT3G44670.2	TIR,NB-ARC,LRR	TNL;complete;18,15,13,1,4,5,10,3,12,7,11,11,11,11,11,11,11,11,11,11	Disease resistance protein (TIR-NBS-LRR class) family
AT3G44400.2	TIR,NB-ARC,LRR	TNL;complete;18,15,13,1,4,5,10,3,12,7,11,11,11,11,11,11,11	Disease resistance protein (TIR-NBS-LRR class) family
AT3G28890.2	LRR	N/A;partial;9,9,9,9,11,11,11,11,11,11	receptor like protein 43
AT4G27220.1	NB-ARC,LRR	CNL;partial;1,6,4,5,10,3,12,2,7,9,11,9,11,11,11,11	NB-ARC domain-containing disease resistance protein
AT4G23440.1	TIR	Not Found	Disease resistance protein (TIR-NBS class)
AT4G16940.1	TIR,NB-ARC,LRR	TNL;partial;15,13,1,4,5,3,7,11,11,11,11,11,11,11,11,9,11,9,11	Disease resistance protein (TIR-NBS-LRR class) family
AT4G16930.1	TIR	Not Found	Toll-Interleukin-Resistance (TIR) domain-containing protein
AT4G16920.1	TIR,NB-ARC,LRR	TNL;complete;18,15,13,1,4,5,3,12,7,9,11,11,11,11,11,11,11,11,11,11,11,11,11,9,11	Disease resistance protein (TIR-NBS-LRR class) family
AT4G16950.1	TIR,NB-ARC,LRR	TNL;complete;18,15,13,1,4,5,3,12,7,9,11,11,11,11,11,11,11,11,11,11,11,11,11,9,11	Disease resistance protein (TIR-NBS-LRR class) family
AT4G16860.1	TIR,NB-ARC,LRR	TNL;complete;18,15,13,1,4,5,3,12,7,11,11,11,11,11,11,11,11,11,11,9,11	Disease resistance protein (TIR-NBS-LRR class) family
AT4G27190.1	NB-ARC,LRR	CNL;partial;1,6,4,5,10,3,12,2,7,9,11,9,11,11,11,11	NB-ARC domain-containing disease resistance protein
AT4G16900.1	TIR,NB-ARC,LRR	TNL;complete;18,15,13,1,4,5,3,12,7,11,11,11,11,11,11,11,11,9,11	Disease resistance protein (TIR-NBS-LRR class) family
AT4G16890.1	TIR,NB-ARC,LRR	TNL;complete;18,15,13,1,4,5,3,12,7,11,11,11,11,11,11,11,11,9,11,11,9,11,11,11,9,11,11,9,11	disease resistance protein (TIR-NBS-LRR class), putative
AT4G16960.1	TIR,NB-ARC,LRR	TNL;complete;18,15,13,1,4,5,3,7,11,11,11,11,11,11,11,11,9,11,9,11	Disease resistance protein (TIR-NBS-LRR class) family
AT4G23510.1	TIR	TNL;partial;18,15,13,18,15	Disease resistance protein (TIR-NBS-LRR class) family
AT4G23515.1	TIR	TNL;partial;18,15,13,17	Toll-Interleukin-Resistance (TIR) domain family protein
AT4G16990.1	TIR,NB-ARC	TNL;partial;18,15,13,1,4,5,10,3,12	disease resistance protein (TIR-NBS class), putative
AT4G19470.1	LRR	N/A;partial;9,11,11	Leucine-rich repeat (LRR) family protein
AT4G19500.1	TIR,NB-ARC,LRR	TNL;complete;18,15,13,1,4,5,10,3,15,13,1,4,5,10,3,11,11,11,9,11	nucleoside-triphosphatases;transmembrane receptors;nucleotide binding;ATP binding
AT4G19510.1	TIR,NB-ARC,LRR	TNL;complete;18,15,13,1,4,5,10,3,7,11,11,11,9,11,11,9,9,11,11	Disease resistance protein (TIR-NBS-LRR class)
AT4G19060.1	NB-ARC	TNL;partial;1,4,5	P-loop containing nucleoside triphosphate hydrolases superfamily protein
AT4G19050.1	NB-ARC,LRR	N/A;complete;1,16,3,12,11,11,9,11,11,9,11,9,11,11,9,11,11,9,11,11,9,11,11	NB-ARC domain-containing disease resistance protein
AT4G08450.1	TIR,NB-ARC,LRR	TNL;complete;18,13,1,4,5,3,12,7,11,11,11,11,11,11,11,11,9,11	Disease resistance protein (TIR-NBS-LRR class) family
AT4G19520.1	TIR,NB-ARC,LRR	TNL;complete;18,15,13,1,4,5,10,3,12,7,11,11,11,9,11,9,11,11,9,11,11,9,11,11,9,11,18,15,13	disease resistance protein (TIR-NBS-LRR class) family
AT4G13810.1	LRR	N/A;partial;11,11,11,9,9,11,11,11	receptor like protein 47
AT4G36150.1	TIR,NB-ARC,LRR	TNL;complete;18,15,13,1,4,5,10,3,12,7,9,11,11,11,9,11,11,9,11,11,19	Disease resistance protein (TIR-NBS-LRR class) family
AT4G36140.1	TIR,NB-ARC,LRR	TNL;complete;18,15,13,1,4,5,18,15,13,1,4,5,10,3,7,11,11,9,11,11,11	disease resistance protein (TIR-NBS-LRR class), putative
AT4G09420.1	TIR,NB-ARC	TNL;partial;15,13,1,4,5,10,3	Disease resistance protein (TIR-NBS class)
AT4G09430.1	TIR,NB-ARC,LRR	TNL;complete;18,15,13,1,4,5,10,3,12,7,11,11,9,9,11,9,11	Disease resistance protein (TIR-NBS-LRR class) family
AT4G14370.1	TIR,NB-ARC,LRR	TNL;partial;15,13,1,4,5,10,3,12,7,9,11,11,11,11,9,11,9,11	Disease resistance protein (TIR-NBS-LRR class) family
AT4G09360.1	NB-ARC,LRR	TNL;partial;1,4,5,10,12,11,9,9,11,11,11,11	NB-ARC domain-containing disease resistance protein
AT4G26090.1	NB-ARC,LRR	CNL;partial;1,6,4,5,10,3,12,2,7,11,9,9,11,11,11,11,11,11,11	NB-ARC domain-containing disease resistance protein
AT4G12020.1	NB-ARC,LRR	TNL;partial;3,18,13,1,4,5,9,11,11,11,11,9,11,11,9	protein kinase family protein
AT4G12010.1	TIR,NB-ARC,LRR	TNL;complete;18,15,13,1,4,5,10,3,12,16,7,11,11,11,9,11,11,9,9,9,11,11	Disease resistance protein (TIR-NBS-LRR class) family
AT4G19530.1	TIR,NB-ARC,LRR	TNL;complete;18,15,13,1,4,10,3,7,11,11,11,11,9,11,11,9,11	disease resistance protein (TIR-NBS-LRR class) family
AT4G04110.1	TIR	TNL;partial;18,15,13	Toll-Interleukin-Resistance (TIR) domain family protein
AT4G19910.1	TIR	TNL;partial;18,15,13	Toll-Interleukin-Resistance (TIR) domain family protein
AT4G19920.1	TIR	TNL;partial;18,15,13	Toll-Interleukin-Resistance (TIR) domain family protein
AT4G19925.1	TIR	TNL;partial;18,15,13	Toll-Interleukin-Resistance (TIR) domain family protein
AT4G10780.1	NB-ARC,LRR	CNL;partial;1,6,4,5,10,3,12,2,7,11,11,9,9,11,11,11,11	LRR and NB-ARC domains-containing disease resistance protein
AT4G11340.1	TIR	N/A;partial;18,15,18	Disease resistance protein (TIR-NBS-LRR class) family
AT4G33300.1	NB-ARC,LRR	Not Found	ADR1-like 1
AT4G11170.1	TIR,NB-ARC,LRR	TNL;complete;18,15,13,1,4,5,10,3,12,7,9,11,11,11,11,9,11,11	Disease resistance protein (TIR-NBS-LRR class) family
AT4G19510.2	TIR,NB-ARC,LRR	TNL;complete;18,15,13,1,4,5,10,3,7,11,11,11,9,11,11,9	Disease resistance protein (TIR-NBS-LRR class)
AT4G16950.2	TIR,NB-ARC,LRR	TNL;complete;18,15,13,1,4,5,3,12,7,9,11,11,11,11,11,11,11,11,11,11,11,11,11,9,11	Disease resistance protein (TIR-NBS-LRR class) family
AT4G16990.3	NB-ARC	TNL;partial;1,4,5,10,3,12	disease resistance protein (TIR-NBS class), putative
AT4G16990.2	TIR,NB-ARC	TNL;partial;18,15,13,1,4,5,10,3,12	disease resistance protein (TIR-NBS class), putative

AT4G16990.4	NB-ARC	TNL;partial;1,4,5,10,3,12	disease resistance protein (TIR-NBS class), putative
AT4G33300.2	NB-ARC,LRR	Not Found	ADR1-like 1
AT4G12020.2	NB-ARC,LRR	TNL;partial;3,18,13,1,4,5,9,11,11,11,11,9,11,11,9	protein kinase family protein
AT4G16990.5	NB-ARC	TNL;partial;1,4,5,10,3,12	disease resistance protein (TIR-NBS class), putative
AT4G12020.3	NB-ARC,LRR	TNL;partial;3,18,13,1,4,5,9,11,11,11,11,9,11,11,9	protein kinase family protein
AT4G13810.2	LRR	N/A;partial;11,11,9,9,11,11,11	receptor like protein 47
AT4G19500.2	TIR,NB-ARC,LRR	TNL;partial;15,13,1,4,5,10,3,11,11,11,9,11	nucleoside-triphosphatases;transmembrane receptors;nucleotide binding;ATP binding
AT5G56220.1	NB-ARC	TNL;partial;9,1,4,5,3	P-loop containing nucleoside triphosphate hydrolases superfamily protein
AT5G66910.1	NB-ARC,LRR	CNL;partial;1,6,4,3,12,2,11,11,11,11	Disease resistance protein (CC-NBS-LRR class) family
AT5G66900.1	NB-ARC,LRR	Not Found	Disease resistance protein (CC-NBS-LRR class) family
AT5G44510.1	TIR,NB-ARC,LRR	TNL;complete;18,15,13,1,4,5,10,3,12,7,11,11,11,11,11,11,11,11,11,11,11,11,11	target of AVRB operation1
AT5G66630.1	NB-ARC	CNL;partial;1,6,4	DA1-related protein 5
AT5G58120.1	TIR,NB-ARC,LRR	TNL;complete;18,15,13,1,4,5,10,3,12,7,11,11,11,11,11	Disease resistance protein (TIR-NBS-LRR class) family
AT5G48780.1	TIR,NB-ARC	TNL;partial;18,15,13,1,4,5,10,3,1,4,5,10,3	disease resistance protein (TIR-NBS class)
AT5G48770.1	TIR,NB-ARC,LRR	TNL;complete;18,15,13,1,4,5,10,3,12,7,11,11,11,9,11,11,9,11,11	Disease resistance protein (TIR-NBS-LRR class) family
AT5G35450.1	NB-ARC,LRR	CNL;complete;17,16,17,1,6,4,5,10,3,12,2,8,7,9,11,11	Disease resistance protein (CC-NBS-LRR class) family
AT5G47260.1	NB-ARC,LRR	CNL;partial;17,1,4,10,3,2,7,11,9,11,11,11,11,11,1	ATP binding;GTP binding;nucleotide binding;nucleoside-triphosphatases
AT5G47280.1	NB-ARC,LRR	CNL;partial;1,4,10,3,2,7,9,11,11,11,11	ADR1-like 3
AT5G47250.1	NB-ARC,LRR	CNL;partial;1,6,4,5,10,3,12,2,8,7,11,9,11,11,11	LRR and NB-ARC domains-containing disease resistance protein
AT5G45440.1	NB-ARC	TNL;partial;1,4,5,3	P-loop containing nucleoside triphosphate hydrolases superfamily protein
AT5G40060.1	NB-ARC,LRR	TNL;partial;1,4,5,10,3,12,7,11,11,11,11,9,11,11,11,9,11	Disease resistance protein (NBS-LRR class) family
AT5G43470.1	NB-ARC,LRR	CNL;complete;17,16,17,1,6,4,5,10,3,12,2,8,7,9,11	Disease resistance protein (CC-NBS-LRR class) family
AT5G40090.1	NB-ARC	TNL;partial;1,4,5,10,3	Disease resistance protein (TIR-NBS class)
AT5G40100.1	TIR,NB-ARC,LRR	TNL;complete;18,15,13,1,4,5,10,3,12,7,11,11,9,11,11,11,9,11	Disease resistance protein (TIR-NBS-LRR class) family
AT5G17680.1	TIR,NB-ARC,LRR	TNL;complete;18,15,13,1,4,5,10,3,12,7,9,11,11,11,9,11,11,11,9,11,11,9,9,9,9,11,11	disease resistance protein (TIR-NBS-LRR class), putative
AT5G49140.1	TIR,NB-ARC,LRR	TNL;complete;18,15,13,1,4,5,3,12,7,11,11,11,11,9,11,11	Disease resistance protein (TIR-NBS-LRR class) family
AT5G04720.1	NB-ARC,LRR	Not Found	ADR1-like 2
AT5G46260.1	TIR,NB-ARC,LRR	TNL;complete;18,15,13,1,4,5,10,3,12,7,11,11,11,11,9,11,11,11,9	disease resistance protein (TIR-NBS-LRR class) family
AT5G46270.1	TIR,NB-ARC,LRR	TNL;complete;18,15,13,1,4,5,10,3,12,7,11,11,11,11,9,11,11,11,9,11	Disease resistance protein (TIR-NBS-LRR class) family
AT5G40910.1	TIR,NB-ARC,LRR	TNL;complete;18,15,13,1,4,5,10,3,12,7,9,11,11,11,11,9,11	Disease resistance protein (TIR-NBS-LRR class) family
AT5G48620.1	NB-ARC,LRR	CNL;complete;17,16,17,1,6,4,5,10,3,12,2,8,7,9,11	Disease resistance protein (CC-NBS-LRR class) family
AT5G63020.1	NB-ARC,LRR	CNL;partial;17,1,6,4,5,10,3,12,2,7,11,9,11,11,11,11	Disease resistance protein (CC-NBS-LRR class) family
AT5G22690.1	TIR,NB-ARC,LRR	TNL;complete;18,15,13,1,4,5,10,3,12,7,11,11,11,11,9,11	Disease resistance protein (TIR-NBS-LRR class) family
AT5G51630.1	TIR,NB-ARC,LRR	TNL;complete;18,15,13,1,4,5,3,12,7,11,11,11,11,9,11,11,11,11,9,11	Disease resistance protein (TIR-NBS-LRR class) family
AT5G41540.1	TIR,NB-ARC,LRR	TNL;complete;18,15,13,1,4,5,3,7,9,11,11,11,11,9,9,11,11	Disease resistance protein (TIR-NBS-LRR class) family
AT5G41550.1	TIR,NB-ARC,LRR	TNL;complete;18,15,13,1,4,5,10,3,12,7,9,11,11,11,11,9,9,11	Disease resistance protein (TIR-NBS-LRR class) family
AT5G46470.1	TIR,NB-ARC,LRR	TNL;complete;18,15,13,1,4,5,3,12,7,9,11,11,11,11,11,11,11,9	disease resistance protein (TIR-NBS-LRR class) family
AT5G46490.2	TIR,NB-ARC,LRR	TNL;complete;18,15,13,1,4,5,10,3,12,7,11,11,11,11,9	Disease resistance protein (TIR-NBS-LRR class) family
AT5G46450.1	TIR,NB-ARC,LRR	TNL;complete;18,15,13,1,4,5,10,3,12,7,11,11,11,11,9,11,11,11,9,11	Disease resistance protein (TIR-NBS-LRR class) family
AT5G46520.1	TIR,NB-ARC,LRR	TNL;complete;18,15,13,1,4,5,3,7,11,11,11,9,11,11,11,9,11	Disease resistance protein (TIR-NBS-LRR class) family
AT5G46500.1	Not Found	Not Found	BEST Arabidopsis thaliana protein match is: disease resistance protein (TIR-NBS-LRR class)
AT5G46510.1	TIR,NB-ARC,LRR	TNL;complete;18,15,13,1,4,5,3,7,11,11,11,9,11,11,11,9,11	Disease resistance protein (TIR-NBS-LRR class) family
AT5G38850.1	TIR,NB-ARC,LRR	TNL;complete;18,13,1,4,5,10,3,7,11,11,11,11,9,11,9,11	Disease resistance protein (TIR-NBS-LRR class)
AT5G45510.1	LRR	N/A;partial;1,8,11,11,9,11,9,11,9,11,11,9,11,11,11	Leucine-rich repeat (LRR) family protein
AT5G45490.1	NB-ARC	TNL;partial;1,4,5	P-loop containing nucleoside triphosphate hydrolases superfamily protein
AT5G41740.1	TIR,NB-ARC,LRR	TNL;complete;18,15,1,4,5,10,3,12,9,11,11,11,11,9,11	Disease resistance protein (TIR-NBS-LRR class) family
AT5G41750.1	TIR,NB-ARC,LRR	TNL;complete;18,15,13,1,4,5,10,3,12,11,11,11,11,9,9,11	Disease resistance protein (TIR-NBS-LRR class) family
AT5G18350.1	TIR,NB-ARC,LRR	TNL;complete;18,15,13,1,4,5,10,3,12,7,11,11,11,11,11,11,9,11,9,11,11,11	Disease resistance protein (TIR-NBS-LRR class) family
AT5G18360.1	TIR,NB-ARC,LRR	TNL;complete;18,13,1,4,5,10,3,12,7,11,11,11,11,9,11,9,11,11	Disease resistance protein (TIR-NBS-LRR class) family
AT5G38350.1	NB-ARC,LRR	TNL;partial;1,4,5,10,3,12,7,11,11,11,11,9,11,11	Disease resistance protein (NBS-LRR class) family
AT5G45000.1	TIR	TNL;partial;18,15,13,18,15,13	Disease resistance protein (TIR-NBS-LRR class) family
AT5G43020.1	LRR	N/A;partial;9,11,12	Leucine-rich repeat protein kinase family protein
AT5G05400.1	NB-ARC,LRR	CNL;partial;17,1,6,4,5,3,12,2,7,11,9,9,11,11,11	LRR and NB-ARC domains-containing disease resistance protein
AT5G38340.1	TIR,NB-ARC,LRR	TNL;complete;18,15,13,1,4,5,10,3,12,7,11,11,11,11,11,9,11,11	Disease resistance protein (TIR-NBS-LRR class) family
AT5G43740.1	NB-ARC,LRR	CNL;partial;1,6,4,5,10,3,12,2,7,11,11,9,9,11,11,11	Disease resistance protein (CC-NBS-LRR class) family
AT5G17890.1	NB-ARC,LRR	TNL;complete;18,13,1,4,5,10,3,11,11,9,11,11,11	DA1-related protein 4
AT5G17880.1	TIR,NB-ARC,LRR	TNL;complete;18,15,13,1,4,5,10,3,7,11,11,11,11,9,11,11,9,11,11,19	disease resistance protein (TIR-NBS-LRR class)
AT5G11250.1	TIR,NB-ARC,LRR	TNL;complete;18,15,13,1,4,5,10,3,12,7,11,11,11,11,11,11,11,11,11,11,11,9,11,11	Disease resistance protein (TIR-NBS-LRR class)
AT5G45060.1	TIR,NB-ARC,LRR	TNL;complete;18,15,13,1,4,5,10,3,12,7,11,11,9,11,11,11,11,11	Disease resistance protein (TIR-NBS-LRR class) family
AT5G45070.1	TIR	TNL;partial;18,15,13	phloem protein 2-A8
AT5G45080.1	TIR	TNL;partial;18,15,13	phloem protein 2-A6
AT5G43730.1	NB-ARC,LRR	CNL;partial;1,6,4,5,10,3,12,2,7,11,9,9,11,11,11	Disease resistance protein (CC-NBS-LRR class) family
AT5G45050.1	NB-ARC,LRR	TNL;complete;18,13,1,4,5,10,3,7,11,11,9,11,11,9,7	Disease resistance protein (TIR-NBS-LRR class)
AT5G36930.1	TIR,NB-ARC,LRR	TNL;complete;18,15,13,1,4,5,10,3,12,7,11,11,11,9,12,9,11	Disease resistance protein (TIR-NBS-LRR class) family
AT5G45230.1	TIR,NB-ARC,LRR	TNL;complete;18,15,13,1,4,5,10,3,12,7,11,11,11,11,9,11,11,9,11,11,11,19	Disease resistance protein (TIR-NBS-LRR class) family
AT5G44900.1	TIR	TNL;partial;18,15,13	Toll-Interleukin-Resistance (TIR) domain family protein
AT5G18370.1	TIR,NB-ARC,LRR	TNL;complete;18,15,13,1,4,5,10,3,12,7,11,11,11,11,11,9,11,9,11,11	Disease resistance protein (TIR-NBS-LRR class) family
AT5G44870.1	TIR,NB-ARC,LRR	TNL;complete;18,15,13,1,4,5,10,3,7,9,9,11,11,11,9,11,11,9,11,11	Disease resistance protein (TIR-NBS-LRR class) family
AT5G45240.1	TIR,NB-ARC,LRR	TNL;partial;18,15,13,1,4,5,10,11,11,11,11,9,9,11	Disease resistance protein (TIR-NBS-LRR class)
AT5G45250.1	TIR,NB-ARC,LRR	TNL;complete;18,15,13,1,4,5,10,3,12,7,11,11,11,9,11,11,11,11,11	Disease resistance protein (TIR-NBS-LRR class) family
AT5G45260.1	NB-ARC,LRR	TNL;complete;18,15,1,4,5,10,3,12,7,9,11,11,9,11,9,11,9,12,7	Disease resistance protein (TIR-NBS-LRR class)
AT5G17970.1	TIR,NB-ARC,LRR	TNL;complete;18,15,13,1,4,5,10,3,7,11,11,11,11,11,9,11	Disease resistance protein (TIR-NBS-LRR class) family
AT5G44920.1	TIR	TNL;partial;18,15,13	Toll-Interleukin-Resistance (TIR) domain family protein
AT5G45210.1	TIR,NB-ARC,LRR	TNL;complete;18,15,13,1,4,5,10,3,12,11,9	Disease resistance protein (TIR-NBS-LRR class) family
AT5G45200.1	TIR,NB-ARC,LRR	TNL;complete;18,15,13,1,4,5,10,3,11,11,11,9,11,11,9,9,11,11	Disease resistance protein (TIR-NBS-LRR class) family
AT5G45220.1	TIR	TNL;partial;18,15,13,18,15,13	Disease resistance protein (TIR-NBS-LRR class) family
AT5G44910.1	TIR	TNL;partial;18,15,13	Toll-Interleukin-Resistance (TIR) domain family protein
AT5G45050.2	TIR,NB-ARC,LRR	TNL;complete;18,13,1,4,5,10,3,7,11,11,9,11,11,9,7	Disease resistance protein (TIR-NBS-LRR class)
AT5G43740.2	NB-ARC,LRR	CNL;partial;1,6,4,5,10,3,12,2,7,11,11,9,9,11,11,11	Disease resistance protein (CC-NBS-LRR class) family
AT5G51630.2	TIR,NB-ARC,LRR	TNL;partial;15,13,1,4,5,3,12,7,11,11,11,11,9,11,11,11,11,9,11	Disease resistance protein (TIR-NBS-LRR class) family
AT5G46490.1	TIR,NB-ARC	TNL;partial;18,15,13,1,4,5	Disease resistance protein (TIR-NBS-LRR class) family
AT5G43470.2	NB-ARC,LRR	CNL;complete;17,16,17,1,6,4,5,10,3,12,2,8,7,9,11	Disease resistance protein (CC-NBS-LRR class) family
AT5G41750.2	TIR,NB-ARC,LRR	TNL;complete;18,15,13,1,4,5,10,3,12,11,11,11,11,9,9,11	Disease resistance protein (TIR-NBS-LRR class) family
AT5G44920.2	TIR	TNL;partial;18,15,13	Toll-Interleukin-Resistance (TIR) domain family protein
AT5G38344.1	TIR	TNL;partial;18,15,13	Toll-Interleukin-Resistance (TIR) domain family protein
AT5G45260.2	NB-ARC,LRR	TNL;complete;18,15,1,4,5,10,3,12,7,9,11,11,9,11,9,11,9,12,7	Disease resistance protein (TIR-NBS-LRR class)
AT5G45490.2	NB-ARC	TNL;partial;1,4,5	P-loop containing nucleoside triphosphate hydrolases superfamily protein
AT5G45510.2	LRR	N/A;partial;1,8,11,11,9,11,9,11,9,11,11,9,11,11,11	Leucine-rich repeat (LRR) family protein
AT5G36930.2	TIR,NB-ARC,LRR	TNL;complete;18,15,13,1,4,5,10,3,12,7,11,11,11,9,12,9,11	Disease resistance protein (TIR-NBS-LRR class) family
AT5G41740.2	TIR,NB-ARC,LRR	TNL;complete;18,15,1,4,5,10,3,12,9,11,11,11,11,9,11	Disease resistance protein (TIR-NBS-LRR class) family
AT5G51630.3	TIR,NB-ARC,LRR	TNL;complete;18,15,13,4,5,3,12,7,11,11,11,11,9,11,11,11,11,9,11	Disease resistance protein (TIR-NBS-LRR class) family
AT5G48780.2	TIR,NB-ARC	TNL;partial;18,15,13,1,4,5,10,3,1,4,5,10	disease resistance protein (TIR-NBS class)

Supplementary Table 2: NLR annotation of *Brachypodium distachyon* proteins using PFAM domains, NLR-Parser and text search

Gene ID	HMMER Scan	NLR Parser	Annotation1 (homology *Arabidopsis*)	Annotation2 (homology rice)
Bradi0013s00100.1.p	NB-ARC,LRR	CNL;partial;17,1,6,4,5,3,12,2,8,7,9,11,9,19,11,11,11	NB-ARC domain-containing disease resistance protein	NBS-LRR disease resistance protein, putative, expressed
Bradi0022s00100.1.p	NB-ARC	Not Found	NB-ARC domain-containing disease resistance protein	disease resistance protein RGA1, putative, expressed
Bradi0022s00400.1.p	NB-ARC,LRR	CNL;complete;17,16,1,6,4,5,10,3,12,2,7,9,11,11,11,11,11,11,11,11	LRR and NB-ARC domains-containing disease resistance protein	disease resistance protein RGA2, putative, expressed
Bradi1g00227.2.p	NB-ARC	CNL;partial;17,16,1,6,4,5,10,3,12,2,8	NB-ARC domain-containing disease resistance protein	disease resistance protein RPM1, putative, expressed
Bradi1g00227.3.p	NB-ARC	CNL;partial;17,16,1,6,4,5,10,3,12,2,8	NB-ARC domain-containing disease resistance protein	disease resistance protein RPM1, putative, expressed
Bradi1g00232.1.p	LRR	N/A;partial;9,9,9,11,11	NB-ARC domain-containing disease resistance protein	disease resistance protein RPM1, putative, expressed
Bradi1g00237.2.p	NB-ARC,LRR	CNL;complete;17,16,1,6,4,5,10,3,2,8,7,9,11	NB-ARC domain-containing disease resistance protein	pollen signalling protein with adenylyl cyclase activity, putative, expressed
Bradi1g00278.1.p	NB-ARC,LRR	CNL;partial;17,16,1,4,5,10,12,2,7,9,9,11,9,19,11,11	NB-ARC domain-containing disease resistance protein	pollen signalling protein with adenylyl cyclase activity, putative, expressed
Bradi1g00284.1.p	LRR	N/A;partial;19,11,11,11,9,11	LRR and NB-ARC domains-containing disease resistance protein	NBS-LRR disease resistance protein, putative, expressed
Bradi1g00960.3.p	NB-ARC,LRR	CNL;complete;17,16,1,6,4,5,10,3,12,2,8,7,9,9,11,11,11,11	LRR and NB-ARC domains-containing disease resistance protein	NBS-LRR type disease resistance protein Hom-F, putative, expressed
Bradi1g01250.1.p	NB-ARC,LRR	CNL;partial;17,16,1,6,4,5,10,3,12,2,8,7,9,9	NB-ARC domain-containing disease resistance protein	NB-ARC domain containing protein, expressed
Bradi1g01250.2.p	NB-ARC,LRR	CNL;partial;17,16,1,6,4,5,10,3,12,2,8,7,9,9	NB-ARC domain-containing disease resistance protein	NB-ARC domain containing protein, expressed
Bradi1g01250.3.p	NB-ARC,LRR	CNL;partial;17,16,1,6,4,5,10,3,12,2,8,7,9,9	NB-ARC domain-containing disease resistance protein	NB-ARC domain containing protein, expressed
Bradi1g01250.4.p	NB-ARC,LRR	CNL;partial;17,16,1,6,4,5,10,3,12,2,8,7,9,9	NB-ARC domain-containing disease resistance protein	NB-ARC domain containing protein, expressed
Bradi1g01257.1.p	NB-ARC,LRR	CNL;complete;17,16,14,1,6,4,5,3,12,2,8,7,11,9,9	NB-ARC domain-containing disease resistance protein	MLA6 protein, putative, expressed
Bradi1g01377.1.p	NB-ARC,LRR	CNL;complete;17,16,1,6,4,5,10,3,12,2,8,7,11,9,9	NB-ARC domain-containing disease resistance protein	MLA6 protein, putative, expressed
Bradi1g01387.2.p	NB-ARC	CNL;complete;17,16,1,6,4,5,10,3,12,2,8,7,11,9,9	NB-ARC domain-containing disease resistance protein	MLA6 protein, putative, expressed
Bradi1g01387.3.p	NB-ARC	CNL;complete;17,16,1,6,4,5,10,3,12,2,8,7,11,9,9	NB-ARC domain-containing disease resistance protein	MLA6 protein, putative, expressed
Bradi1g01397.1.p	NB-ARC	CNL;partial;17,16,1,6,4,5,10	NB-ARC domain-containing disease resistance protein	resistance protein, putative, expressed
Bradi1g01407.1.p	NB-ARC,LRR	CNL;complete;17,16,14,1,6,4,5,10,3,12,2,8,7,11,9,9,11	NB-ARC domain-containing disease resistance protein	MLA6 protein, putative, expressed
Bradi1g01442.2.p	LRR	N/A;partial;9,11,11,11,11,11,12,17,11,11,11,11,12	Leucine-rich repeat protein kinase family protein	receptor-like protein kinase 2 precursor, putative, expressed
Bradi1g01550.2.p	NB-ARC,LRR	Not Found	NB-ARC domain-containing disease resistance protein	resistance protein, putative, expressed
Bradi1g01555.1.p	NB-ARC	CNL;partial;17,16,4,5	NB-ARC domain-containing disease resistance protein	expressed protein
Bradi1g08240.1.p	LRR	N/A;partial;11,11,11,11,9,11	receptor-like protein kinase 2	phytosulfokine receptor precursor, putative, expressed
Bradi1g14050.1.p	LRR	N/A;partial;9,11,11	Leucine-rich repeat (LRR) family protein	uncharacterized protein At4g06744 precursor, putative, expressed
Bradi1g14250.1.p	Not Found	N/A;partial;9,11	RNI-like superfamily protein	F-box/LRR domain containing protein, putative, expressed
Bradi1g14250.2.p	Not Found	N/A;partial;9,11	RNI-like superfamily protein	F-box/LRR domain containing protein, putative, expressed
Bradi1g14250.3.p	Not Found	N/A;partial;9,11	RNI-like superfamily protein	F-box/LRR domain containing protein, putative, expressed
Bradi1g14250.4.p	Not Found	N/A;partial;9,11	RNI-like superfamily protein	F-box/LRR domain containing protein, putative, expressed
Bradi1g14250.5.p	Not Found	N/A;partial;9,11	RNI-like superfamily protein	F-box/LRR domain containing protein, putative, expressed
Bradi1g14250.6.p	Not Found	N/A;partial;9,11	RNI-like superfamily protein	F-box/LRR domain containing protein, putative, expressed
Bradi1g14250.7.p	Not Found	N/A;partial;9,11	RNI-like superfamily protein	F-box/LRR domain containing protein, putative, expressed
Bradi1g14250.8.p	Not Found	N/A;partial;9,11	RNI-like superfamily protein	F-box/LRR domain containing protein, putative, expressed
Bradi1g14250.9.p	Not Found	N/A;partial;9,11	RNI-like superfamily protein	F-box/LRR domain containing protein, putative, expressed
Bradi1g15650.1.p	NB-ARC,LRR	CNL;complete;16,1,4,5,10,3,2,8,7,9,9,19,11,11,11,11,11	LRR and NB-ARC domains-containing disease resistance protein	NBS-LRR type disease resistance protein Rps1-k-1, putative, expressed
Bradi1g17705.1.p	Not Found	Not Found	Ribonuclease H-like superfamily protein	NBS-LRR disease resistance protein, putative, expressed
Bradi1g20750.1.p	LRR	N/A;partial;11,9,11,11,11,11,11	Leucine-rich receptor-like protein kinase family protein	expressed protein
Bradi1g22500.1.p	NB-ARC,LRR	CNL;partial;17,16,1,6,4,5,10,3,12,2,8,7,9,9	NB-ARC domain-containing disease resistance protein	NBS-LRR type disease resistance protein, putative, expressed
Bradi1g22500.2.p	NB-ARC,LRR	CNL;partial;17,16,1,6,4,5,10,3,12,2,8,7,9,9	NB-ARC domain-containing disease resistance protein	NBS-LRR type disease resistance protein, putative, expressed
Bradi1g24230.1.p	LRR	N/A;partial;9,11,12	polygalacturonase inhibiting protein 1	polygalacturonase inhibitor 1 precursor, putative, expressed
Bradi1g24368.1.p	TIR	N/A;partial;18,15	Toll-Interleukin-Resistance (TIR) domain family protein	expressed protein
Bradi1g24377.1.p	TIR	N/A;partial;18,15	Toll-Interleukin-Resistance (TIR) domain family protein	expressed protein
Bradi1g24593.1.p	Not Found	Not Found	RNA-directed DNA polymerase (reverse transcriptase)-related family protein	NBS-LRR disease resistance protein, putative, expressed
Bradi1g27757.2.p	NB-ARC,LRR	CNL;complete;17,16,1,4,5,3,12,2,8,9,9,11,9,9,19,11,11,11	NB-ARC domain-containing disease resistance protein	NBS-LRR disease resistance protein, putative, expressed
Bradi1g27757.3.p	NB-ARC,LRR	CNL;complete;17,16,1,4,5,3,12,2,8,9,9,11,9,9,19,11,11,11	NB-ARC domain-containing disease resistance protein	NBS-LRR disease resistance protein, putative, expressed
Bradi1g27770.1.p	NB-ARC,LRR	CNL;partial;1,6,5,10,3,12,2,8,7,9,9,11,9,11,11,11,11,11	NB-ARC domain-containing disease resistance protein	disease resistant protein, identical, putative, expressed
Bradi1g27770.2.p	NB-ARC,LRR	CNL;partial;1,6,5,10,3,12,2,8,7,9,9,11,9,11,11,11,11,11	NB-ARC domain-containing disease resistance protein	disease resistant protein, identical, putative, expressed
Bradi1g29267.1.p	NB-ARC,LRR	Not Found	NB-ARC domain-containing disease resistance protein	resistance protein, putative, expressed
Bradi1g29267.2.p	NB-ARC,LRR	Not Found	NB-ARC domain-containing disease resistance protein	resistance protein, putative, expressed
Bradi1g29352.1.p	NB-ARC	CNL;partial;17,16,4,5,1,4,5,1,6,4,5,3,2,8,17,9,9	NB-ARC domain-containing disease resistance protein	resistance protein, putative, expressed
Bradi1g29360.2.p	NB-ARC,LRR	CNL;partial;17,6,4,5,4,2,8,11,9	Disease resistance protein (CC-NBS-LRR class) family	resistance protein, putative, expressed
Bradi1g29370.2.p	NB-ARC	CNL;partial;1,6,4,3,2,8,9	Disease resistance protein (CC-NBS-LRR class) family	resistance protein, putative, expressed
Bradi1g29381.1.p	NB-ARC	CNL;partial;17,6,4,5,1,6,4,5,3,2,8,11,9	NB-ARC domain-containing disease resistance protein	resistance protein, putative, expressed
Bradi1g29422.1.p	NB-ARC	CNL;partial;17,16,1,6,4	NB-ARC domain-containing disease resistance protein	disease resistance RPP8-like protein 3, putative, expressed
Bradi1g29427.3.p	NB-ARC,LRR	CNL;complete;17,16,1,6,4,5,10,3,12,2,8,7,9,11,9,11	Disease resistance protein (CC-NBS-LRR class) family	disease resistance RPP8-like protein 3, putative, expressed
Bradi1g29427.4.p	NB-ARC,LRR	CNL;complete;17,16,1,6,4,5,10,3,12,2,8,7,9,11,9,11	Disease resistance protein (CC-NBS-LRR class) family	disease resistance RPP8-like protein 3, putative, expressed
Bradi1g29427.5.p	NB-ARC,LRR	CNL;complete;17,16,1,6,4,5,10,3,12,2,8,7,9,11,9,11	Disease resistance protein (CC-NBS-LRR class) family	disease resistance RPP8-like protein 3, putative, expressed
Bradi1g29435.1.p	NB-ARC	CNL;partial;17,1,5,10,12,12,2,8,7	NB-ARC domain-containing disease resistance protein	disease resistance RPP8-like protein 3, putative, expressed
Bradi1g29441.2.p	NB-ARC,LRR	CNL;partial;1,6,4,5,10,3,12,2,8,7,9,9,11	NB-ARC domain-containing disease resistance protein	disease resistance RPP8-like protein 3, putative, expressed
Bradi1g29446.1.p	NB-ARC,LRR	CNL;complete;17,16,1,6,4,5,10,3,12,2,8,7,9,11,11	NB-ARC domain-containing disease resistance protein	disease resistance RPP8-like protein 3, putative, expressed
Bradi1g29560.1.p	NB-ARC,LRR	CNL;complete;17,16,1,6,4,5,10,3,12,2,8,7,9,9,11,9,11,11,11,11,11	NB-ARC domain-containing disease resistance protein	pollen signalling protein with adenylyl cyclase activity, putative, expressed
Bradi1g29560.2.p	NB-ARC,LRR	CNL;complete;17,16,1,6,4,5,10,3,12,2,8,7,9,9,11,9,11,11,11,11,11	NB-ARC domain-containing disease resistance protein	pollen signalling protein with adenylyl cyclase activity, putative, expressed
Bradi1g29634.2.p	NB-ARC	CNL;partial;14,1,6,4,11,11	LRR and NB-ARC domains-containing disease resistance protein	xa1, putative, expressed
Bradi1g29658.2.p	NB-ARC	CNL;complete;16,1,6,4,5,3,12,2,7,11,11,9,11,11,11,11	LRR and NB-ARC domains-containing disease resistance protein	xa1, putative, expressed
Bradi1g30667.1.p	Not Found	N/A;partial;8,9,11,11,11,11		Leucine Rich Repeat family protein, expressed
Bradi1g31971.1.p	LRR	N/A;partial;9,11,9,11,11,9,11,11,9,11,11	disease resistance family protein / LRR family protein	leucine rich repeat protein, putative, expressed
Bradi1g31971.2.p	LRR	N/A;partial;9,11,11,9,11,11,9,11,11	disease resistance family protein / LRR family protein	leucine rich repeat protein, putative, expressed
Bradi1g33020.1.p	LRR	N/A;partial;11,11,11,11,11,11,9,11,11	receptor like protein 46	Cf2/Cf5 disease resistance protein, putative, expressed
Bradi1g34050.2.p	NB-ARC	Not Found	NB-ARC domain-containing disease resistance protein	NB-ARC domain containing disease resistance protein, putative, expressed
Bradi1g34370.1.p	NB-ARC	Not Found	NB-ARC domain-containing disease resistance protein	expressed protein
Bradi1g34407.1.p	NB-ARC	Not Found	NB-ARC domain-containing disease resistance protein	expressed protein
Bradi1g34430.2.p	NB-ARC,LRR	TNL;partial;1,4,10,9,9,9,11	NB-ARC domain-containing disease resistance protein	Leucine Rich Repeat family protein, expressed
Bradi1g37190.1.p	LRR	N/A;partial;11,11,11,11,9,11,11,11,11,11,11,11,11	Leucine-rich receptor-like protein kinase family protein	receptor-like protein kinase 2 precursor, putative, expressed
Bradi1g41825.1.p	NB-ARC,LRR	CNL;complete;17,16,4,3,12,2,8,7,9,9,9,11,16	NB-ARC domain-containing disease resistance protein	NB-ARC domain containing protein, expressed
Bradi1g42250.2.p	Not Found	Not Found	Disease resistance protein (TIR-NBS-LRR class) family	OsFBX178 - F-box domain containing protein, expressed
Bradi1g44425.1.p	LRR	N/A;partial;11,11,9,9,11,11,12	disease resistance family protein / LRR family protein	leucine rich repeat protein, putative, expressed
Bradi1g44542.1.p	NB-ARC,LRR	CNL;partial;17,16,14,1,6,4,5,10,3,12,2,8,7,9	NB-ARC domain-containing disease resistance protein	NBS-LRR disease resistance protein, putative, expressed
Bradi1g44542.2.p	NB-ARC,LRR	CNL;partial;17,16,14,1,6,4,5,10,3,12,2,8,7,9	NB-ARC domain-containing disease resistance protein	NBS-LRR disease resistance protein, putative, expressed
Bradi1g47130.1.p	LRR	N/A;partial;11,11,11,11,9,11,11,11,9,11,11	Leucine-rich receptor-like protein kinase family protein	leucine-rich repeat receptor protein kinase EXS precursor, putative, expressed
Bradi1g47610.2.p	LRR	N/A;partial;11,9,11,11,11,11,11,11,12	receptor like protein 15	receptor-like protein kinase 5 precursor, putative, expressed
Bradi1g47618.1.p	LRR	N/A;partial;11,11,9,11,11,9,11,12	receptor like protein 21	receptor-like protein kinase 5 precursor, putative, expressed
Bradi1g48747.1.p	NB-ARC,LRR	CNL;complete;17,16,1,4,5,10,3,12,2,8,7,9,9,11,11	NB-ARC domain-containing disease resistance protein	NBS-LRR type disease resistance protein, putative, expressed
Bradi1g49950.1.p	LRR	N/A;partial;11,11,11,11,11,9,11,11,12	ERECTA-like 2	receptor-like protein kinase 5 precursor, putative, expressed
Bradi1g49950.2.p	LRR	N/A;partial;11,11,11,11,11,9,11,11,12	ERECTA-like 2	receptor-like protein kinase 5 precursor, putative, expressed
Bradi1g50407.1.p	NB-ARC,LRR	CNL;complete;17,16,1,6,4,5,3,12,2,8,7,11,9,19,11,11	NB-ARC domain-containing disease resistance protein	NBS-LRR disease resistance protein, putative, expressed
Bradi1g50407.3.p	NB-ARC,LRR	CNL;complete;17,16,1,6,4,5,3,12,2,8,7,11,9,19,11,11	NB-ARC domain-containing disease resistance protein	NBS-LRR disease resistance protein, putative, expressed
Bradi1g50407.4.p	NB-ARC,LRR	CNL;complete;17,16,1,6,4,5,3,12,2,8,7,11,9,19,11,11	NB-ARC domain-containing disease resistance protein	NBS-LRR disease resistance protein, putative, expressed
Bradi1g50407.5.p	NB-ARC,LRR	CNL;complete;17,16,1,6,4,5,3,12,2,8,7,11,9,19,11,11	NB-ARC domain-containing disease resistance protein	NBS-LRR disease resistance protein, putative, expressed
Bradi1g50420.2.p	NB-ARC,LRR	CNL;complete;17,16,1,6,4,5,3,2,8,7,11,9,19,11	NB-ARC domain-containing disease resistance protein	NBS-LRR disease resistance protein, putative, expressed
Bradi1g51320.1.p	NB-ARC,LRR	CNL;partial;17,5,3,2,9,11,9,9	NB-ARC domain-containing disease resistance protein	resistance protein, putative, expressed
Bradi1g51687.1.p	NB-ARC,LRR	CNL;complete;17,16,1,6,4,5,10,3,12,2,8,7,11,9,9,11	NB-ARC domain-containing disease resistance protein	MLA6 protein, putative, expressed
Bradi1g51940.2.p	NB-ARC	Not Found	cell division cycle 48C	AAA family ATPase, putative, expressed
Bradi1g51954.1.p	NB-ARC	CNL;partial;16,1,6,4,5,10,3,12,2,8,7,9	NB-ARC domain-containing disease resistance protein	NB-ARC domain containing protein, expressed
Bradi1g51961.1.p	NB-ARC	CNL;complete;17,16,1,6,4,5,10,3,12,2,8,7,9,11	myb domain protein 92	RGH2B, putative, expressed
Bradi1g51961.2.p	NB-ARC	CNL;complete;17,16,1,6,4,5,10,3,12,2,8,7,9,11	NB-ARC domain-containing disease resistance protein	RGH2B, putative, expressed
Bradi1g51961.3.p	NB-ARC	CNL;complete;17,16,1,6,4,5,10,3,12,2,8,7,9,11	NB-ARC domain-containing disease resistance protein	RGH2B, putative, expressed
Bradi1g51961.4.p	NB-ARC	CNL;complete;17,16,1,6,4,5,10,3,12,2,8,7,9,11	NB-ARC domain-containing disease resistance protein	RGH2B, putative, expressed
Bradi1g52554.1.p	Not Found	Not Found		NBS-LRR type disease resistance protein, putative, expressed
Bradi1g52760.2.p	Not Found	N/A;partial;18,15	Protein phosphatase 2A regulatory B subunit family protein	serine/threonine protein phosphatase 2A 59 kDa regulatory subunit Bgamma isoform, putative, expressed
Bradi1g52760.3.p	Not Found	N/A;partial;18,15	Protein phosphatase 2A regulatory B subunit family protein	serine/threonine protein phosphatase 2A 59 kDa regulatory subunit Bgamma isoform, putative, expressed
Bradi1g52760.4.p	Not Found	N/A;partial;18,15	Protein phosphatase 2A regulatory B subunit family protein	serine/threonine protein phosphatase 2A 59 kDa regulatory subunit Bgamma isoform, putative, expressed

Bradi1g52760.5.p	Not Found	N/A;partial;18,15	Protein phosphatase 2A regulatory B subunit family protein	serine/threonine protein phosphatase 2A 59 kDa regulatory subunit Bgamma isoform, putative, expressed
Bradi1g53930.1.p	NB-ARC	Not Found	NB-ARC domain-containing disease resistance protein	NB-ARC domain containing protein, expressed
Bradi1g53940.2.p	NB-ARC	Not Found		expressed protein
Bradi1g55080.1.p	NB-ARC,LRR	CNL;complete;17,16,1,6,4,5,10,3,12,2,8,7,9,9,11,9,11,11,11	NB-ARC domain-containing disease resistance protein	disease resistance protein RPM1, putative, expressed
Bradi1g55672.1.p	NB-ARC,LRR	CNL;complete;17,16,1,6,4,5,10,3,12,2,8,7,11,9,11	HOPZ-ACTIVATED RESISTANCE 1	resistance protein, putative, expressed
Bradi1g56695.1.p	NB-ARC,LRR	CNL;partial;1,6,4,5,2,7,9,9,9,11,11,11,11,11,11,11,9	LRR and NB-ARC domains-containing disease resistance protein	Leucine Rich Repeat family protein, expressed
Bradi1g57271.1.p	Not Found	CNL;partial;17,2,8,7	HOPZ-ACTIVATED RESISTANCE 1	expressed protein
Bradi1g57831.1.p	Not Found	Not Found	RNA-directed DNA polymerase (reverse transcriptase)-related family protein	NBS-LRR disease resistance protein, putative, expressed
Bradi1g58940.1.p	NB-ARC	Not Found	NB-ARC domain-containing disease resistance protein	expressed protein
Bradi1g58950.1.p	NB-ARC	Not Found	NB-ARC domain-containing disease resistance protein	expressed protein
Bradi1g58965.1.p	NB-ARC	Not Found		expressed protein
Bradi1g58980.1.p	NB-ARC	Not Found	LRR and NB-ARC domains-containing disease resistance protein	expressed protein
Bradi1g59360.1.p	LRR	N/A;partial;9,11,9,11,11,11,11,11	phytosylfokine-alpha receptor 2	phytosulfokine receptor precursor, putative, expressed
Bradi1g59465.1.p	NB-ARC	TNL;partial;1,4,5	P-loop containing nucleoside triphosphate hydrolases superfamily protein	expressed protein
Bradi1g60397.2.p	LRR	N/A;partial;11,9,11	STRUBBELIG-receptor family 7	STRUBBELIG-RECEPTOR FAMILY 6 precursor, putative, expressed
Bradi1g60552.1.p	Not Found	Not Found		NBS-LRR disease resistance protein, putative, expressed
Bradi1g61015.1.p	Not Found	Not Found	Polynucleotidyl transferase, ribonuclease H-like superfamily protein	NBS-LRR disease resistance protein, putative, expressed
Bradi1g63200.1.p	LRR	N/A;partial;11,11,11,11,11,11,9,11,11,11	Leucine-rich repeat protein kinase family protein	receptor-like protein kinase precursor, putative, expressed
Bradi1g67840.1.p	NB-ARC,LRR	CNL;partial;1,4,5,10,3,12,2,7,9,14,11,11,11,11	NB-ARC domain-containing disease resistance protein	NB-ARC/LRR disease resistance protein, putative, expressed
Bradi1g69097.1.p	LRR	N/A;partial;9,11,11,11,11,11,11,12,6	Leucine-rich receptor-like protein kinase family protein	receptor protein kinase CLAVATA1 precursor, putative, expressed
Bradi1g77940.2.p	Not Found	CNL;partial;17,17,16	Plant protein of unknown function (DUF827)	paramyosin, putative, expressed
Bradi1g77940.3.p	Not Found	CNL;partial;17,17,16	Plant protein of unknown function (DUF827)	paramyosin, putative, expressed
Bradi1g78800.2.p	LRR	N/A;partial;17,8,7,9,9	NB-ARC domain-containing disease resistance protein	expressed protein
Bradi1g78926.1.p	NB-ARC	CNL;complete;16,17,1,18,4,5,3,2,8,7,11,11,11	NB-ARC domain-containing disease resistance protein	NBS-LRR disease resistance protein, putative, expressed
Bradi1g79026.1.p	NB-ARC	Not Found	NB-ARC domain-containing disease resistance protein	NB-ARC domain containing protein, expressed
Bradi2g01070.1.p	LRR	N/A;partial;6,9,11,11,9,11,11,11,11	Disease resistance protein (CC-NBS-LRR class) family	Leucine Rich Repeat family protein, expressed
Bradi2g01077.1.p	LRR	N/A;partial;6,11,9,11,11,11,11,11	NB-ARC domain-containing disease resistance protein	Leucine Rich Repeat family protein, expressed
Bradi2g01280.2.p	LRR	N/A;partial;11,11,9,11,11,11,11,11	Disease resistance protein (CC-NBS-LRR class) family	Leucine Rich Repeat family protein, expressed
Bradi2g02160.1.p	LRR	N/A;partial;9,11,11,11,11,11,11,11,11,11,11,9,11,11	receptor like protein 34	verticillium wilt disease resistance protein, putative, expressed
Bradi2g02290.1.p	LRR	N/A;partial;9,11,11	leucine-rich repeat transmembrane protein kinase family protein	inactive receptor kinase At2g26730 precursor, putative, expressed
Bradi2g02863.1.p	Not Found	Not Found		NBS-LRR disease resistance protein, putative, expressed
Bradi2g03009.1.p	NB-ARC	CNL;partial;17,1,6,4	NB-ARC domain-containing disease resistance protein	disease resistance protein, putative, expressed
Bradi2g03012.1.p	NB-ARC,LRR	CNL;partial;3,12,2,8,7,9,11,9,19,11	NB-ARC domain-containing disease resistance protein	NBS-LRR disease resistance protein, putative, expressed
Bradi2g03020.1.p	NB-ARC,LRR	CNL;partial;17,1,6,4,5,3,12,2,8,7,9,11,9,19,11,11	NB-ARC domain-containing disease resistance protein	NBS-LRR disease resistance protein, putative, expressed
Bradi2g03040.1.p	NB-ARC	CNL;partial;17,1,6,4,5,3	NB-ARC domain-containing disease resistance protein	disease resistance protein, putative, expressed
Bradi2g03061.1.p	NB-ARC,LRR	CNL;partial;17,1,6,4,5,3,12,2,8,7,9,11,9,19,11,11,11	NB-ARC domain-containing disease resistance protein	NBS-LRR disease resistance protein, putative, expressed
Bradi2g03061.2.p	NB-ARC,LRR	CNL;partial;17,1,6,4,5,3,12,2,8,7,9,11,9,19,11,11,11	NB-ARC domain-containing disease resistance protein	NBS-LRR disease resistance protein, putative, expressed
Bradi2g03061.3.p	NB-ARC,LRR	CNL;partial;17,1,6,4,5,3,12,2,8,7,9,11,9,19,11,11,11	NB-ARC domain-containing disease resistance protein	NBS-LRR disease resistance protein, putative, expressed
Bradi2g03061.4.p	NB-ARC,LRR	CNL;partial;17,1,6,4,5,3,12,2,8,7,9,11,9,19,11,11,11	NB-ARC domain-containing disease resistance protein	NBS-LRR disease resistance protein, putative, expressed
Bradi2g03191.1.p	NB-ARC,LRR	CNL;complete;17,16,1,6,4,5,10,3,12,8,7,11,9,9,9,9,19,11,11,11	NB-ARC domain-containing disease resistance protein	NBS-LRR disease resistance protein, putative, expressed
Bradi2g03191.2.p	NB-ARC,LRR	CNL;complete;17,16,1,6,4,5,10,3,12,8,7,11,9,9,9,9,19,11,11,11	NB-ARC domain-containing disease resistance protein	NBS-LRR disease resistance protein, putative, expressed
Bradi2g03200.2.p	NB-ARC,LRR	CNL;complete;17,16,1,6,4,5,3,2,8,7,11,9,9,11,9,19,11,11,11	NB-ARC domain-containing disease resistance protein	NBS-LRR disease resistance protein, putative, expressed
Bradi2g03260.1.p	NB-ARC,LRR	CNL;complete;17,16,1,6,4,5,3,12,2,8,7,11,9,9,11,9,19,11,11,11	NB-ARC domain-containing disease resistance protein	NBS-LRR disease resistance protein, putative, expressed
Bradi2g03588.1.p	NB-ARC,LRR	CNL;partial;17,16,14,1,6,4,5,10,3,12,2,8,7,9,9,9	NB-ARC domain-containing disease resistance protein	stripe rust resistance protein Yr10, putative, expressed
Bradi2g03853.1.p	LRR	N/A;partial;9,11,9,11,12,11,19,11,12	receptor like protein 6	hcr2-5D, putative, expressed
Bradi2g03886.1.p	LRR	N/A;partial;9,11,9,9,9,11,11,11,11,11	receptor like protein 6	verticillium wilt disease resistance protein Ve2, putative, expressed
Bradi2g03900.2.p	LRR	N/A;partial;9,9,11,9	receptor like protein 6	resistance protein SlVe1 precursor, putative, expressed
Bradi2g03910.1.p	LRR	N/A;partial;9,9,11,11,11,11	receptor like protein 22	verticillium wilt disease resistance protein Ve2, putative, expressed
Bradi2g03920.2.p	LRR	N/A;partial;9,9,9,9,11,11,11,9,11,11,11,11	receptor like protein 23	verticillium wilt disease resistance protein Ve2, putative, expressed
Bradi2g05611.1.p	NB-ARC	Not Found	NB-ARC domain-containing disease resistance protein	NB-ARC domain containing disease resistance protein, putative, expressed
Bradi2g07306.1.p	Not Found	Not Found		NBS-LRR disease resistance protein, putative, expressed
Bradi2g08990.2.p	Not Found	CNL;partial;17,16,2,8,11,5,17	HOPZ-ACTIVATED RESISTANCE 1	expressed protein
Bradi2g09280.1.p	Not Found	N/A;partial;18,15	high affinity K+ transporter 5	potassium transporter, putative, expressed
Bradi2g09427.1.p	NB-ARC,LRR	CNL;complete;17,16,1,4,5,10,3,12,2,8,7,11,9,9	NB-ARC domain-containing disease resistance protein	NB-ARC domain containing protein, expressed
Bradi2g09434.1.p	NB-ARC	CNL;partial;17,16,1,6,4,5,10,3,12,2,8,7	NB-ARC domain-containing disease resistance protein	RGH2B, putative, expressed
Bradi2g09480.1.p	NB-ARC,LRR	CNL;complete;17,16,1,6,4,5,10,3,2,8,7,9,9,11,9,11,11,11,11,11,11,11,11,19,11,11,11,11	LRR and NB-ARC domains-containing disease resistance protein	disease resistance protein RGA2, putative, expressed
Bradi2g11931.1.p	NB-ARC	CNL;partial;17,16,14,1,6,4,5,10,3,12,2,8,7,9	NB-ARC domain-containing disease resistance protein	NBS-LRR disease resistance protein, putative, expressed
Bradi2g12497.1.p	NB-ARC,LRR	CNL;partial;17,16,1,6,4,5,10,3,12,2,8,7,9,9	NB-ARC domain-containing disease resistance protein	stripe rust resistance protein Yr10, putative, expressed
Bradi2g13865.1.p	Not Found	Not Found	Ribonuclease H-like superfamily protein	NBS-LRR disease resistance protein, putative, expressed
Bradi2g17843.1.p	Not Found	Not Found		NBS-LRR disease resistance protein, putative, expressed
Bradi2g18451.1.p	Not Found	Not Found	Ribonuclease H-like superfamily protein	NBS-LRR disease resistance protein, putative, expressed
Bradi2g18830.2.p	NB-ARC	Not Found	LRR and NB-ARC domains-containing disease resistance protein	expressed protein
Bradi2g18840.1.p	NB-ARC	Not Found	NB-ARC domain-containing disease resistance protein	expressed protein
Bradi2g18851.1.p	NB-ARC	Not Found	LRR and NB-ARC domains-containing disease resistance protein	expressed protein
Bradi2g21360.1.p	NB-ARC,LRR	CNL;complete;17,16,1,6,4,5,10,3,12,2,8,7,9,9,11,11,11,11,11,11,11,11,11	NB-ARC domain-containing disease resistance protein	disease resistance RPP13-like protein 1, putative, expressed
Bradi2g25327.1.p	NB-ARC,LRR	CNL;complete;16,1,6,4,5,10,3,12,2,8,7,9,11,11,11,11,11,11	NB-ARC domain-containing disease resistance protein	vrga1, putative, expressed
Bradi2g26396.1.p	Not Found	Not Found		NBS-LRR disease resistance protein, putative, expressed
Bradi2g26926.1.p	NB-ARC,LRR	CNL;partial;3,2,8,7,11,11,9,19,11	NB-ARC domain-containing disease resistance protein	pollen signalling protein with adenylyl cyclase activity, putative, expressed
Bradi2g27750.1.p	NB-ARC	Not Found	NB-ARC domain-containing disease resistance protein	expressed protein
Bradi2g27912.1.p	Not Found	Not Found	RNA-directed DNA polymerase (reverse transcriptase)-related family protein	NBS-LRR disease resistance protein, putative, expressed
Bradi2g34986.1.p	NB-ARC,LRR	CNL;complete;17,16,1,6,4,5,10,3,2,8,7,9,11,19,11,11,11	NB-ARC domain-containing disease resistance protein	pollen signalling protein with adenylyl cyclase activity, putative, expressed
Bradi2g35767.1.p	NB-ARC	CNL;complete;17,16,1,6,4,5,3,12,2,8,7,19,11,11,11,11,11,11	LRR and NB-ARC domains-containing disease resistance protein	NBS-LRR disease resistance protein, putative, expressed
Bradi2g36030.2.p	NB-ARC,LRR	CNL;complete;16,1,6,4,5,3,12,2,8,7,9,11,19,11,11,11,11,11,11,11,11,11,11	LRR and NB-ARC domains-containing disease resistance protein	NBS-LRR disease resistance protein, putative, expressed
Bradi2g36032.1.p	Not Found	Not Found		NBS-LRR disease resistance protein, putative, expressed
Bradi2g36037.1.p	NB-ARC,LRR	CNL;complete;16,1,6,4,5,3,12,2,8,7,9,11,19,11,11,11,11,11,11,11,11,11,11,11,11,11	LRR and NB-ARC domains-containing disease resistance protein	NBS-LRR disease resistance protein, putative, expressed
Bradi2g36150.1.p	NB-ARC	CNL;partial;1,6,4,5,3,12,2	NB-ARC domain-containing disease resistance protein	NBS-LRR disease resistance protein, putative, expressed
Bradi2g36170.1.p	Not Found	CNL;partial;17,16	NB-ARC domain-containing disease resistance protein	disease resistance protein RGA3, putative, expressed
Bradi2g36173.1.p	NB-ARC	CNL;partial;1,6,4,5,8	NB-ARC domain-containing disease resistance protein	disease resistance protein RGA3, putative, expressed
Bradi2g36176.1.p	LRR	N/A;partial;9,9,11,11,11,9,11,9,11,19,11,11,11,11,11,11	disease resistance protein (TIR-NBS-LRR class), putative	NBS-LRR disease resistance protein, putative, expressed
Bradi2g36179.1.p	NB-ARC	Not Found	NB-ARC domain-containing disease resistance protein	NBS-LRR disease resistance protein, putative, expressed
Bradi2g36182.1.p	LRR	N/A;partial;9,9,11,11,11,11,11,11,11,11,11,11,11,11,11,11,11	disease resistance protein (TIR-NBS-LRR class)	NBS-LRR disease resistance protein, putative, expressed
Bradi2g37166.1.p	NB-ARC,LRR	CNL;complete;17,16,1,6,4,5,10,3,12,2,8,7,11,9,9,9	NB-ARC domain-containing disease resistance protein	MLA6 protein, putative, expressed
Bradi2g37166.2.p	NB-ARC	CNL;complete;17,16,1,6,4,5,10,3,12,2,8,7,11,9,9,9	NB-ARC domain-containing disease resistance protein	MLA6 protein, putative, expressed
Bradi2g37172.2.p	NB-ARC	CNL;partial;1,6,4,5,10,3,12,2,8,7,11,9,9,9,11	NB-ARC domain-containing disease resistance protein	MLA6 protein, putative, expressed
Bradi2g37172.3.p	NB-ARC	CNL;partial;1,6,4,5,10,3,12,2,8,7,11,9,9,9,11	NB-ARC domain-containing disease resistance protein	MLA6 protein, putative, expressed
Bradi2g37191.1.p	NB-ARC,LRR	CNL;complete;17,16,14,1,6,5,10,3,12,2,8,7,11,9,9,9	NB-ARC domain-containing disease resistance protein	MLA6 protein, putative, expressed
Bradi2g37260.1.p	NB-ARC,LRR	Not Found		Leucine Rich Repeat family protein, expressed
Bradi2g37990.1.p	NB-ARC,LRR	CNL;complete;17,16,1,4,5,10,3,12,2,8,7,9,9,9,11	NB-ARC domain-containing disease resistance protein	expressed protein
Bradi2g37990.2.p	NB-ARC,LRR	CNL;partial;4,5,10,3,12,2,8,7,9,9,9,11	NB-ARC domain-containing disease resistance protein	expressed protein
Bradi2g38192.1.p	NB-ARC	CNL;complete;16,1,6,4,5,3,12,2,8,7,9,11,11,11,11	NB-ARC domain-containing disease resistance protein	xa1, putative, expressed
Bradi2g38791.1.p	NB-ARC	CNL;complete;17,16,1,6,4,5,4,5,3,12,2,8,7,11,11,11	Disease resistance protein (CC-NBS-LRR class) family	resistance protein, putative, expressed
Bradi2g38800.2.p	NB-ARC,LRR	CNL;complete;17,16,6,4,5,1,6,4,5,10,3,8,11,11,9,11,11	NB-ARC domain-containing disease resistance protein	NB-ARC domain containing protein, expressed
Bradi2g38810.2.p	NB-ARC,LRR	CNL;complete;17,16,1,6,4,5,1,6,4,5,10,3,12,2,8,7,11,11,9,7,11,11	NB-ARC domain-containing disease resistance protein	resistance protein, putative, expressed
Bradi2g38827.2.p	NB-ARC,LRR	CNL;complete;17,16,6,4,5,10,1,4,5,10,3,12,2,8,7,11,11,9,11,11	NB-ARC domain-containing disease resistance protein	resistance protein, putative, expressed
Bradi2g38838.1.p	NB-ARC	CNL;complete;17,16,1,6,4,1,4,5,10,3,8,7,11,11,9	NB-ARC domain-containing disease resistance protein	NB-ARC domain containing protein, expressed
Bradi2g38900.2.p	NB-ARC,LRR	CNL;partial;17,6,4,5,10,1,6,4,5,2,8,7,11,11,9,11,11	NB-ARC domain-containing disease resistance protein	NB-ARC domain containing protein, expressed
Bradi2g38928.1.p	NB-ARC,LRR	CNL;complete;17,16,1,4,10,1,4,5,3,8,7,9,11,11	Disease resistance protein (CC-NBS-LRR class) family	resistance protein, putative, expressed
Bradi2g38982.1.p	NB-ARC,LRR	CNL;complete;17,16,14,1,6,4,5,10,3,12,2,8,7,11,9,9	Disease resistance protein (CC-NBS-LRR class) family	RGH1A, putative, expressed
Bradi2g38982.2.p	NB-ARC,LRR	CNL;complete;17,16,14,1,6,4,5,10,3,12,2,8,7,11,9,9	Disease resistance protein (CC-NBS-LRR class) family	RGH1A, putative, expressed
Bradi2g38987.4.p	NB-ARC,LRR	CNL;partial;5,10,3,12,2,8,7,9	NB-ARC domain-containing disease resistance protein	RGH1A, putative, expressed
Bradi2g38991.1.p	NB-ARC	CNL;partial;17,16,14,1	NB-ARC domain-containing disease resistance protein	RGH1A, putative, expressed
Bradi2g39067.1.p	NB-ARC,LRR	CNL;complete;16,14,1,6,4,5,10,3,12,2,8,7,11,9,11	Disease resistance protein (CC-NBS-LRR class) family	RGH1A, putative, expressed
Bradi2g39067.2.p	NB-ARC,LRR	CNL;complete;16,14,1,6,4,5,10,3,12,2,8,7,11,9,11	Disease resistance protein (CC-NBS-LRR class) family	RGH1A, putative, expressed

Bradi2g39074.1.p	NB-ARC,LRR	CNL;complete;17,16,14,1,6,4,5,10,3,12,2,8,7,11,9	Disease resistance protein (CC-NBS-LRR class) family	RGH1A, putative, expressed
Bradi2g39091.1.p	NB-ARC	CNL;complete;17,16,14,1,6,4,5,10,3,12,2,8,7,11	Disease resistance protein (CC-NBS-LRR class) family	RGH1A, putative, expressed
Bradi2g39207.1.p	NB-ARC,LRR	CNL;complete;17,16,14,1,6,4,5,10,3,12,2,8,11,9,9	NB-ARC domain-containing disease resistance protein	MLA6 protein, putative, expressed
Bradi2g39247.1.p	NB-ARC,LRR	CNL;complete;17,16,14,1,6,4,5,10,3,12,2,8,7,9,11,20	NB-ARC domain-containing disease resistance protein	RGH1A, putative, expressed
Bradi2g39393.1.p	NB-ARC,LRR	CNL;complete;17,16,14,1,6,4,5,10,3,12,2,8,7,9,11,20	NB-ARC domain-containing disease resistance protein	RGH1A, putative, expressed
Bradi2g39430.1.p	LRR	N/A;partial;16,9,11,11,9,11,11,9,11,11,11,11	Disease resistance protein (TIR-NBS-LRR class) family	Leucine Rich Repeat family protein, expressed
Bradi2g39440.1.p	LRR	N/A;partial;12,9,11,9,9,11,9,11,11,9,11,11,11,14	disease resistance protein (TIR-NBS-LRR class)	Leucine Rich Repeat family protein, expressed
Bradi2g39460.1.p	LRR	CNL;partial;2,9,11,9,9,11,9,11,11,9,11,11,11,11,11	disease resistance protein (TIR-NBS-LRR class)	Leucine Rich Repeat family protein, expressed
Bradi2g39517.2.p	NB-ARC	CNL;complete;17,16,1,6,4,5,1,6,4,5,10,3,12,2,8,7,11,11,9	Disease resistance protein (CC-NBS-LRR class) family	resistance protein, putative, expressed
Bradi2g39517.3.p	NB-ARC	CNL;complete;17,16,1,6,4,5,1,6,4,5,10,3,12,2,8,7,11,11,9	Disease resistance protein (CC-NBS-LRR class) family	resistance protein, putative, expressed
Bradi2g39537.1.p	NB-ARC	CNL;partial;14,1,6,4,5,10,3,12,2,8,7,9,11,9,9,9	NB-ARC domain-containing disease resistance protein	resistance protein, putative, expressed
Bradi2g39547.1.p	NB-ARC,LRR	CNL;partial;1,6,4,5,10,3,12,2,8,7,11,11,9,10	Disease resistance protein (CC-NBS-LRR class) family	resistance protein, putative, expressed
Bradi2g39560.1.p	NB-ARC	CNL;partial;17,16,6,4,5	NB-ARC domain-containing disease resistance protein	pib, putative, expressed
Bradi2g39575.1.p	LRR	Not Found	disease resistance protein (TIR-NBS-LRR class)	resistance protein LR10, putative, expressed
Bradi2g39622.1.p	NB-ARC	CNL;complete;17,16,14,4,5,10,3,12,2,8,7,11,9,9	NB-ARC domain-containing disease resistance protein	resistance protein LR10, putative, expressed
Bradi2g39648.1.p	NB-ARC	CNL;complete;17,16,14,1,6,4,5,10,3,12,2,8,7,11,9,9	NB-ARC domain-containing disease resistance protein	resistance protein LR10, putative, expressed
Bradi2g39657.1.p	Not Found	CNL;partial;17,16	NB-ARC domain-containing disease resistance protein	disease resistance protein, putative, expressed
Bradi2g39666.1.p	NB-ARC,LRR	CNL;partial;1,6,4,10,3,12,2,8,7,11,9,9	NB-ARC domain-containing disease resistance protein	resistance protein, putative, expressed
Bradi2g39847.1.p	NB-ARC,LRR	CNL;complete;17,16,1,6,4,5,10,3,12,2,8,7,9,9,9,11,11	NB-ARC domain-containing disease resistance protein	expressed protein
Bradi2g40936.1.p	Not Found	Not Found	RNA-directed DNA polymerase (reverse transcriptase)-related family protein	NBS-LRR disease resistance protein, putative, expressed
Bradi2g41651.1.p	Not Found	Not Found	RNA-directed DNA polymerase (reverse transcriptase)-related family protein	NBS-LRR disease resistance protein, putative, expressed
Bradi2g41921.1.p	LRR	Not Found	disease resistance protein (TIR-NBS-LRR class)	NBS-LRR disease resistance protein, putative, expressed
Bradi2g41930.2.p	NB-ARC	CNL;partial;17,16,6,5,10,3,2,8	NB-ARC domain-containing disease resistance protein	disease resistance protein RGA4, putative, expressed
Bradi2g42812.1.p	LRR	N/A;partial;9,11,11	disease resistance family protein / LRR family protein	expressed protein
Bradi2g42820.2.p	LRR	N/A;partial;9,11,11,11,11,11,11,11,11,11,11,11	disease resistance family protein / LRR family protein	expressed protein
Bradi2g42825.1.p	LRR	N/A;partial;9,11,11,11,11,11,11,11,11	disease resistance family protein / LRR family protein	leucine rich repeat protein, putative, expressed
Bradi2g42880.2.p	LRR	N/A;partial;9,9,11,11,11	disease resistance family protein / LRR family protein	leucine rich repeat protein, putative, expressed
Bradi2g47256.1.p	LRR	N/A;partial;11,11,11,11,11,11,9,11	Leucine-rich repeat protein kinase family protein	receptor protein kinase, putative, expressed
Bradi2g47256.2.p	LRR	N/A;partial;11,11,11,11,11,11,9,11	Leucine-rich repeat protein kinase family protein	receptor protein kinase, putative, expressed
Bradi2g48467.1.p	NB-ARC	Not Found	NB-ARC domain-containing disease resistance protein	NB-ARC domain containing protein, expressed
Bradi2g48480.2.p	NB-ARC	Not Found	NB-ARC domain-containing disease resistance protein	expressed protein
Bradi2g48487.1.p	NB-ARC	Not Found	NB-ARC domain-containing disease resistance protein	NB-ARC domain containing protein, expressed
Bradi2g48530.2.p	LRR	N/A;partial;9,11,11	Leucine-rich repeat transmembrane protein kinase protein	protein kinase domain containing protein, expressed
Bradi2g48880.1.p	Not Found	N/A;partial;9,11	F-box/RNI-like superfamily protein	OsFBX23 - F-box domain containing protein, expressed
Bradi2g50590.2.p	NB-ARC	Not Found	Disease resistance protein (TIR-NBS class)	AP003256-AK101847 - NBS/LRR genes that are S-rich,divergent TIR, divergent NBS, expressed
Bradi2g50590.3.p	NB-ARC	Not Found	Disease resistance protein (TIR-NBS class)	AP003256-AK101847 - NBS/LRR genes that are S-rich,divergent TIR, divergent NBS, expressed
Bradi2g50590.4.p	NB-ARC	Not Found	Disease resistance protein (TIR-NBS class)	AP003256-AK101847 - NBS/LRR genes that are S-rich,divergent TIR, divergent NBS, expressed
Bradi2g51807.1.p	NB-ARC,LRR	CNL;complete;16,1,6,4,5,10,3,2,8,7,9,9,11,11,11	LRR and NB-ARC domains-containing disease resistance protein	rp1, putative, expressed
Bradi2g52150.1.p	NB-ARC,LRR	CNL;partial;17,1,4,5,10,3,12,2,7,11,9,9,9,11,11,11,11,11	LRR and NB-ARC domains-containing disease resistance protein	disease resistance protein RPS2, putative, expressed
Bradi2g52430.1.p	NB-ARC	CNL;partial;1,6,4,5	HOPZ-ACTIVATED RESISTANCE 1	NB-ARC domain containing protein, expressed
Bradi2g52437.2.p	NB-ARC	CNL;partial;17,12,2,8,7,11,11,11	Leucine-rich repeat (LRR) family protein	disease resistance protein, putative, expressed
Bradi2g52450.1.p	NB-ARC,LRR	CNL;partial;1,5,3,12,2,7,9,11,11,9,11,11,11	Disease resistance protein (CC-NBS-LRR class) family	NB-ARC/LRR disease resistance protein, putative, expressed
Bradi2g52450.2.p	NB-ARC,LRR	CNL;partial;1,5,3,12,2,7,9,11,11,9,11,11	Disease resistance protein (CC-NBS-LRR class) family	NB-ARC/LRR disease resistance protein, putative, expressed
Bradi2g52450.3.p	NB-ARC,LRR	CNL;partial;1,5,3,12,2,7,9,11,11,9,11,11	Disease resistance protein (CC-NBS-LRR class) family	NB-ARC/LRR disease resistance protein, putative, expressed
Bradi2g52840.1.p	NB-ARC,LRR	CNL;complete;17,16,1,6,4,5,10,3,12,2,8,7,9,9,11,19,11,11,11,19,11,11,11,11,11	NB-ARC domain-containing disease resistance protein	disease resistance RPP13-like protein 1, putative, expressed
Bradi2g52890.1.p	LRR	N/A;partial;9,11	Leucine-rich repeat (LRR) family protein	BRASSINOSTEROID INSENSITIVE 1-associated receptor kinase 1 precursor, putative, expressed
Bradi2g57534.1.p	NB-ARC,LRR	CNL;complete;17,16,14,1,6,4,5,10,3,12,2,8,7,11,9,9	NB-ARC domain-containing disease resistance protein	stripe rust resistance protein Yr10, putative, expressed
Bradi2g58780.2.p	LRR	N/A;partial;9,11,11,9,17,11,11	Leucine-rich repeat transmembrane protein kinase	leucine-rich repeat receptor protein kinase EXS precursor, putative, expressed
Bradi2g58780.3.p	LRR	N/A;partial;9,11,11,9,17,11,11	Leucine-rich repeat transmembrane protein kinase	leucine-rich repeat receptor protein kinase EXS precursor, putative, expressed
Bradi2g58906.1.p	Not Found	Not Found	Ribonuclease H-like superfamily protein	NBS-LRR disease resistance protein, putative, expressed
Bradi2g59310.1.p	NB-ARC,LRR	CNL;complete;17,16,1,6,4,10,3,12,2,8,7,9,9,11,11	NB-ARC domain-containing disease resistance protein	NB-ARC domain containing protein, expressed
Bradi2g60230.2.p	NB-ARC,LRR	CNL;complete;17,16,1,6,4,5,10,3,2,8,7,9,9,11,11,11,11,11,11,11,11,11,11,11,11,11,11,11,11,11	disease resistance protein (TIR-NBS-LRR class)	disease resistance protein RGA4, putative, expressed
Bradi2g60230.3.p	NB-ARC,LRR	CNL;complete;17,16,1,6,4,5,10,3,2,8,7,9,9,11,11,11,11,11,11,11,11,11,11,11,11,11,11,11,11,11	disease resistance protein (TIR-NBS-LRR class)	disease resistance protein RGA4, putative, expressed
Bradi2g60230.4.p	NB-ARC,LRR	CNL;complete;17,16,1,6,4,5,10,3,2,8,7,9,9,11,11,11,11,11,11,11,11,11,11,11,11,11,11,11,11,11	disease resistance protein (TIR-NBS-LRR class)	disease resistance protein RGA4, putative, expressed
Bradi2g60230.5.p	NB-ARC,LRR	CNL;complete;17,16,1,6,4,5,10,3,2,8,7,9,9,11,11,11,11,11,11,11,11,11,11,11,11,11,11,11,11,11	disease resistance protein (TIR-NBS-LRR class)	disease resistance protein RGA4, putative, expressed
Bradi2g60250.1.p	NB-ARC,LRR	CNL;partial;1,4,5,10,3,2,8,7,9,9,11,11,11,11,11,11,11,11,11,11,19,11,11,11,11,9,11,11	disease resistance protein (TIR-NBS-LRR class)	disease resistance protein RGA4, putative, expressed
Bradi2g60255.1.p	Not Found	Not Found		NBS-LRR disease resistance protein, putative, expressed
Bradi2g60260.2.p	NB-ARC,LRR	CNL;complete;17,16,1,6,4,5,10,3,2,8,7,9,9,9,11,11,11,11,11,11,11,11,11,9,11,11,11,11,11,11,11,11,11	disease resistance protein (TIR-NBS-LRR class)	disease resistance protein RGA4, putative, expressed
Bradi2g60260.3.p	NB-ARC,LRR	CNL;complete;17,16,1,6,4,5,10,3,2,8,7,9,9,9,11,11,11,11,11,11,11,11,11,9,11,11,11,11,11,11,11,11,11	disease resistance protein (TIR-NBS-LRR class)	disease resistance protein RGA4, putative, expressed
Bradi2g60418.1.p	NB-ARC,LRR	CNL;complete;17,16,14,1,6,4,5,10,3,12,2,8,7,11,9,9,9	NB-ARC domain-containing disease resistance protein	RGH1A, putative, expressed
Bradi2g60427.1.p	NB-ARC	CNL;partial;17,16,14,1	NB-ARC domain-containing disease resistance protein	RGH1A, putative, expressed
Bradi2g60430.1.p	NB-ARC,LRR	CNL;partial;10,3,12,2,8,7,11,9,11	Disease resistance protein (CC-NBS-LRR class) family	RGH1A, putative, expressed
Bradi2g60434.1.p	NB-ARC,LRR	CNL;complete;16,14,1,6,4,5,10,3,12,2,8,7,11,9,9,9	NB-ARC domain-containing disease resistance protein	RGH1A, putative, expressed
Bradi2g60434.2.p	NB-ARC,LRR	CNL;complete;16,14,1,6,4,5,10,3,12,2,8,7,11,9,9,9	NB-ARC domain-containing disease resistance protein	RGH1A, putative, expressed
Bradi2g61247.1.p	LRR	N/A;partial;11,11,11,11,9,11	Leucine-rich receptor-like protein kinase family protein	expressed protein
Bradi2g61456.1.p	LRR	N/A;partial;9,11,11	receptor like protein 26	verticillium wilt disease resistance protein, putative, expressed
Bradi2g61474.1.p	LRR	N/A;partial;9,11,10,11,11,9,20,11,11,11	receptor like protein 34	verticillium wilt disease resistance protein, putative, expressed
Bradi2g61510.1.p	NB-ARC	TNL;partial;1,4,5,1,4,5,10,3	P-loop containing nucleoside triphosphate hydrolases superfamily protein	disease resistance protein RPS2, putative, expressed
Bradi2g61510.2.p	NB-ARC	TNL;partial;1,4,5,1,4,5,10,3	P-loop containing nucleoside triphosphate hydrolases superfamily protein	disease resistance protein RPS2, putative, expressed
Bradi2g61520.1.p	NB-ARC,LRR	CNL;partial;1,4,5,10,3,2,8,7,9,11,11,11,11,11,11	NB-ARC domain-containing disease resistance protein	NB-ARC/LRR disease resistance protein, putative, expressed
Bradi2g61920.1.p	Not Found	N/A;partial;9,11	ubiquitin-protein ligases	Leucine Rich Repeat family protein, expressed
Bradi3g00741.1.p	NB-ARC,LRR	CNL;complete;17,16,1,6,5,10,3,12,2,8,7,9,9,11,20	NB-ARC domain-containing disease resistance protein	NBS-LRR disease resistance protein, putative, expressed
Bradi3g00757.1.p	NB-ARC	CNL;complete;17,16,14,1,6,4,5,10,3,12,2,8,7,9,11,20	NB-ARC domain-containing disease resistance protein	NBS-LRR disease resistance protein, putative, expressed
Bradi3g00890.2.p	LRR	N/A;partial;10,9,11,9,11,11,11	NB-ARC domain-containing disease resistance protein	Leucine Rich Repeat family protein, expressed
Bradi3g00926.1.p	LRR	N/A;partial;11,9,11,11,11	NB-ARC domain-containing disease resistance protein	Leucine Rich Repeat family protein, expressed
Bradi3g01230.1.p	LRR	N/A;partial;9,11,11,11,11,11,11,11,11,11,11,11	Leucine-rich repeat transmembrane protein kinase family protein	receptor protein kinase CLAVATA1 precursor, putative, expressed
Bradi3g01577.1.p	LRR	N/A;partial;11,9,11,11,11,11,11,11	phytosulfokin receptor 1	phytosulfokine receptor precursor, putative, expressed
Bradi3g03465.1.p	NB-ARC	CNL;complete;17,16,18,1,6,4,5,10,3,12,2,8,7,12,9,9,11	NB-ARC domain-containing disease resistance protein	RGH1A, putative, expressed
Bradi3g03587.1.p	NB-ARC	CNL;partial;17,16,14,1,6,4,5,10,3,12,2,8,7,9	HOPZ-ACTIVATED RESISTANCE 1	NBS-LRR disease resistance protein, putative, expressed
Bradi3g03597.1.p	NB-ARC,LRR	CNL;partial;17,14,1,6,4,5,10,3,12,2,8,7,9,19	NB-ARC domain-containing disease resistance protein	NBS-LRR disease resistance protein, putative, expressed
Bradi3g03874.1.p	NB-ARC,LRR	CNL;complete;17,16,14,1,6,4,5,10,3,12,2,8,7,9,11,9	NB-ARC domain-containing disease resistance protein	NBS-LRR disease resistance protein, putative, expressed
Bradi3g03878.1.p	NB-ARC	CNL;partial;17,16,14,1,6,4,5,10,3,12,2,8,7,9	NB-ARC domain-containing disease resistance protein	NBS-LRR disease resistance protein, putative, expressed
Bradi3g03878.2.p	NB-ARC	CNL;partial;17,16,14,1,6,4,5,10,3,12,2,8,7,9	NB-ARC domain-containing disease resistance protein	NBS-LRR disease resistance protein, putative, expressed
Bradi3g03878.3.p	NB-ARC	CNL;partial;17,16,14,1,6,4,5,10,3,12,2,8,7,9	NB-ARC domain-containing disease resistance protein	NBS-LRR disease resistance protein, putative, expressed
Bradi3g03878.4.p	NB-ARC	CNL;partial;10,3,12,2,8,7,9	NB-ARC domain-containing disease resistance protein	NBS-LRR disease resistance protein, putative, expressed
Bradi3g03882.1.p	NB-ARC,LRR	CNL;complete;17,16,14,1,6,4,5,10,3,12,2,8,7,9,11	NB-ARC domain-containing disease resistance protein	NBS-LRR disease resistance protein, putative, expressed
Bradi3g03935.1.p	NB-ARC	CNL;partial;4,5,3,12,2,8,7,11,12,11	NB-ARC domain-containing disease resistance protein	NBS-LRR disease resistance protein, putative, expressed
Bradi3g04187.1.p	LRR	N/A;partial;11,11,11,11,9,11,11,11	Leucine-rich receptor-like protein kinase family protein	phytosulfokine receptor precursor, putative, expressed
Bradi3g04240.1.p	LRR	N/A;partial;12,11,12,11,11,9,11,11,11,11	receptor like protein 2	phytosulfokine receptor precursor, putative, expressed
Bradi3g04350.2.p	LRR	N/A;partial;12,11,9,9,11	receptor like protein 2	phytosulfokine receptor precursor, putative, expressed
Bradi3g05292.1.p	LRR	N/A;partial;9,9,11,11,11,11,11,11,11,11,11,12	disease resistance family protein / LRR family protein	leucine rich repeat protein, putative, expressed
Bradi3g05470.2.p	NB-ARC,LRR	CNL;partial;17,16,1,6,3,12,2,8,7,9,9	NB-ARC domain-containing disease resistance protein	NB-ARC domain containing protein, expressed
Bradi3g05480.1.p	NB-ARC,LRR	CNL;partial;17,16,1,6,4,3,2,7,9,9	Disease resistance protein (CC-NBS-LRR class) family	NB-ARC domain containing protein, expressed
Bradi3g06980.2.p	LRR	N/A;partial;9,11,11,11,11,11,11,11,11,11	Leucine-rich repeat transmembrane protein kinase	leucine-rich repeat receptor protein kinase EXS precursor, putative, expressed
Bradi3g06980.3.p	LRR	N/A;partial;9,11,11,11,11,11,11,11,11,11	Leucine-rich repeat transmembrane protein kinase	leucine-rich repeat receptor protein kinase EXS precursor, putative, expressed
Bradi3g06980.4.p	LRR	N/A;partial;9,11,11,11,11,11,11,11,11,11	Leucine-rich repeat transmembrane protein kinase	leucine-rich repeat receptor protein kinase EXS precursor, putative, expressed
Bradi3g07315.2.p	NB-ARC	Not Found	HOPZ-ACTIVATED RESISTANCE 1	NB-ARC domain containing disease resistance protein, putative, expressed
Bradi3g07580.2.p	NB-ARC	TNL;partial;1,4,5,3	Disease resistance protein (CC-NBS-LRR class) family	NB-ARC domain containing protein, expressed
Bradi3g07580.3.p	NB-ARC	TNL;partial;1,4,5,3	Disease resistance protein (CC-NBS-LRR class) family	NB-ARC domain containing protein, expressed
Bradi3g07580.4.p	NB-ARC	TNL;partial;1,4,5,3	Disease resistance protein (CC-NBS-LRR class) family	NB-ARC domain containing protein, expressed
Bradi3g07775.1.p	NB-ARC	CNL;partial;17,6,4,5,1,6,4,5,3,2,8,11,9	NB-ARC domain-containing disease resistance protein	resistance protein, putative, expressed
Bradi3g08917.2.p	LRR	N/A;partial;9,11,11,11	Leucine-rich repeat transmembrane protein kinase	leucine-rich repeat-containing protein kinase family protein, putative, expressed
Bradi3g08917.3.p	LRR	N/A;partial;9,11,11,11	Leucine-rich repeat transmembrane protein kinase	leucine-rich repeat-containing protein kinase family protein, putative, expressed

Bradi3g08917.4.p	LRR	N/A;partial;9,11,11,11	Leucine-rich repeat transmembrane protein kinase	leucine-rich repeat-containing protein kinase family protein, putative, expressed
Bradi3g08917.5.p	LRR	N/A;partial;9,11,11,11	Leucine-rich repeat transmembrane protein kinase	leucine-rich repeat-containing protein kinase family protein, putative, expressed
Bradi3g08917.6.p	LRR	N/A;partial;9,11,11,11	Leucine-rich repeat transmembrane protein kinase	leucine-rich repeat-containing protein kinase family protein, putative, expressed
Bradi3g09611.1.p	LRR	N/A;partial;11,11,9,11,11,12,11,11	disease resistance family protein / LRR family protein	leucine rich repeat protein, putative, expressed
Bradi3g10370.2.p	NB-ARC,LRR	CNL;complete;16,1,6,4,5,3,12,2,8,7,9,11,11,11,11,11	NB-ARC domain-containing disease resistance protein	disease resistance protein RGA2, putative, expressed
Bradi3g10370.3.p	NB-ARC,LRR	CNL;partial;16,1,6,4,5,3,12,2,8,7,9	NB-ARC domain-containing disease resistance protein	disease resistance protein RGA2, putative, expressed
Bradi3g10370.4.p	NB-ARC,LRR	CNL;partial;16,1,6,4,5,3,12,2,8,7,9	NB-ARC domain-containing disease resistance protein	disease resistance protein RGA2, putative, expressed
Bradi3g14033.1.p	NB-ARC	CNL;complete;17,16,1,6,4,5,10,3,12,2,8,7,11,9	HOPZ-ACTIVATED RESISTANCE 1	resistance protein LR10, putative, expressed
Bradi3g14033.2.p	NB-ARC	CNL;partial;17,16,1,6,4,5,10,3,12,2,8,7	HOPZ-ACTIVATED RESISTANCE 1	resistance protein LR10, putative, expressed
Bradi3g14540.2.p	LRR	Not Found	Disease resistance protein (TIR-NBS-LRR class)	Leucine Rich Repeat family protein, expressed
Bradi3g14560.2.p	LRR	N/A;partial;11,9,11,11	Disease resistance protein (TIR-NBS-LRR class) family	Leucine Rich Repeat family protein, expressed
Bradi3g14917.1.p	NB-ARC	CNL;partial;11,1,6,4,5,10,3,12,2,8,7,9,11,11,11,11,11,11,11,11	NB-ARC domain-containing disease resistance protein	NBS-LRR disease resistance protein, putative, expressed
Bradi3g14917.2.p	NB-ARC	CNL;partial;1,6,4,5,10,3,12,2,8,7,9,11,11,11,11,11,11,11,11	NB-ARC domain-containing disease resistance protein	NBS-LRR disease resistance protein, putative, expressed
Bradi3g14928.1.p	NB-ARC	CNL;partial;17,1,6,4,5,3,12,2,8,7,9,11,11,11	NB-ARC domain-containing disease resistance protein	NBS-LRR disease resistance protein, putative, expressed
Bradi3g15075.1.p	NB-ARC,LRR	CNL;complete;16,1,6,4,5,10,3,11,11,11,11,11,11,11,11,11,11	LRR and NB-ARC domains-containing disease resistance protein	NBS-LRR disease resistance protein, putative, expressed
Bradi3g15277.2.p	NB-ARC,LRR	CNL;partial;6,4,5,10,3,12,2,8,7,11,9,9	HOPZ-ACTIVATED RESISTANCE 1	resistance protein LR10, putative, expressed
Bradi3g15277.3.p	NB-ARC,LRR	CNL;partial;6,4,5,10,3,12,2,8,7,11,9,9	HOPZ-ACTIVATED RESISTANCE 1	resistance protein LR10, putative, expressed
Bradi3g15277.4.p	NB-ARC,LRR	CNL;partial;6,4,5,10,3,12,2,8,7,11,9,9	HOPZ-ACTIVATED RESISTANCE 1	resistance protein LR10, putative, expressed
Bradi3g15592.1.p	NB-ARC,LRR	CNL;complete;17,16,14,1,6,4,5,10,3,12,2,8,7,11,9,9	NB-ARC domain-containing disease resistance protein	disease resistance protein RPM1, putative, expressed
Bradi3g15593.1.p	NB-ARC,LRR	CNL;complete;17,16,14,1,6,5,10,3,12,2,8,7,11,9,11	HOPZ-ACTIVATED RESISTANCE 1	disease resistance protein RPM1, putative, expressed
Bradi3g15593.2.p	NB-ARC,LRR	CNL;partial;5,10,3,12,2,8,7,11,9,11	NB-ARC domain-containing disease resistance protein	NB-ARC domain containing protein, expressed
Bradi3g15594.1.p	NB-ARC,LRR	CNL;complete;17,16,14,1,6,4,5,10,3,12,2,8,7,11,9,9	HOPZ-ACTIVATED RESISTANCE 1	disease resistance protein RPM1, putative, expressed
Bradi3g15594.2.p	NB-ARC,LRR	CNL;complete;17,16,14,1,6,4,5,10,3,12,2,8,7,11,9,9	HOPZ-ACTIVATED RESISTANCE 1	disease resistance protein RPM1, putative, expressed
Bradi3g15594.3.p	NB-ARC,LRR	CNL;complete;17,16,14,1,6,4,5,10,3,12,2,8,7,11,9,9	HOPZ-ACTIVATED RESISTANCE 1	disease resistance protein RPM1, putative, expressed
Bradi3g15595.1.p	NB-ARC,LRR	CNL;partial;5,10,3,12,2,8,7,11,9,9	NB-ARC domain-containing disease resistance protein	disease resistance protein RPM1, putative, expressed
Bradi3g15596.1.p	Not Found	CNL;partial;17,16,14,6	NB-ARC domain-containing disease resistance protein	disease resistance protein RPM1, putative, expressed
Bradi3g15980.2.p	NB-ARC	Not Found	LRR and NB-ARC domains-containing disease resistance protein	expressed protein
Bradi3g15990.1.p	NB-ARC	Not Found	LRR and NB-ARC domains-containing disease resistance protein	expressed protein
Bradi3g16000.1.p	NB-ARC	Not Found	LRR and NB-ARC domains-containing disease resistance protein	expressed protein
Bradi3g17756.3.p	LRR	N/A;partial;11,9,11	Leucine-rich repeat transmembrane protein kinase	SHR5-receptor-like kinase, putative, expressed
Bradi3g17756.4.p	LRR	N/A;partial;11,9,11	Leucine-rich repeat transmembrane protein kinase	SHR5-receptor-like kinase, putative, expressed
Bradi3g17756.5.p	Not Found	N/A;partial;9,11	Leucine-rich repeat transmembrane protein kinase	SHR5-receptor-like kinase, putative, expressed
Bradi3g18400.2.p	LRR	N/A;partial;11,11,9,11,11,11,11,11,12	disease resistance family protein / LRR family protein	leucine rich repeat protein, putative, expressed
Bradi3g18972.1.p	Not Found	Not Found		NBS-LRR disease resistance protein, putative, expressed
Bradi3g19650.1.p	LRR	N/A;partial;9,11,9,9,11,12	Leucine-rich repeat protein kinase family protein	BRASSINOSTEROID INSENSITIVE 1 precursor, putative, expressed
Bradi3g19967.2.p	NB-ARC,LRR	CNL;partial;17,1,6,4,5,3,12,2,8,7,9,11,11,11	NB-ARC domain-containing disease resistance protein	NBS-LRR disease resistance protein, putative, expressed
Bradi3g19967.3.p	NB-ARC,LRR	CNL;partial;17,1,6,4,5,3,12,2,8,7,9,11,11,11	NB-ARC domain-containing disease resistance protein	NBS-LRR disease resistance protein, putative, expressed
Bradi3g21190.2.p	NB-ARC	Not Found		expressed protein
Bradi3g21210.1.p	NB-ARC	Not Found	NB-ARC domain-containing disease resistance protein	expressed protein
Bradi3g21400.1.p	LRR	N/A;partial;11,11,11,12,11,9,11,11,11,11	BRI1-like 2	serine/threonine-protein kinase BRI1-like 2 precursor, putative, expressed
Bradi3g22520.1.p	NB-ARC,LRR	CNL;complete;17,16,1,6,4,5,10,3,12,2,8,7,17,9,9,9,11,11,11	NB-ARC domain-containing disease resistance protein	disease resistance protein RPM1, putative, expressed
Bradi3g22538.1.p	NB-ARC,LRR	CNL;partial;17,16,1,6,4,2,8,7,17,9,9,11	NB-ARC domain-containing disease resistance protein	disease resistance protein RPM1, putative, expressed
Bradi3g26160.1.p	LRR	N/A;partial;9,11,11,11,11,11,11	F-box family protein	OsFBL52 - F-box domain and LRR containing protein, expressed
Bradi3g28592.1.p	NB-ARC,LRR	CNL;complete;16,1,6,4,5,10,3,12,8,7,9,9,9,11,9,19,11,11,11,9	NB-ARC domain-containing disease resistance protein	NB-ARC domain containing protein, expressed
Bradi3g29642.1.p	Not Found	Not Found	RNA-directed DNA polymerase (reverse transcriptase)-related family protein	NBS-LRR disease resistance protein, putative, expressed
Bradi3g32276.2.p	LRR	N/A;partial;11,11,11,11,11,9,11,11	disease resistance family protein / LRR family protein	leucine rich repeat protein, putative, expressed
Bradi3g34457.1.p	LRR	N/A;partial;11,11,9,11,11,11	PEP1 receptor 1	receptor-like protein kinase precursor, putative, expressed
Bradi3g34457.2.p	LRR	N/A;partial;11,11,9,11,11,11	PEP1 receptor 1	receptor-like protein kinase precursor, putative, expressed
Bradi3g34697.2.p	NB-ARC,LRR	CNL;complete;17,16,1,6,4,5,10,3,12,2,8,7,9,11	NB-ARC domain-containing disease resistance protein	resistance protein LR10, putative, expressed
Bradi3g34697.3.p	NB-ARC,LRR	CNL;complete;17,16,1,6,4,5,10,3,12,2,8,7,9,11	NB-ARC domain-containing disease resistance protein	resistance protein LR10, putative, expressed
Bradi3g34697.4.p	NB-ARC,LRR	CNL;complete;17,16,1,6,4,5,10,3,12,2,8,7,9,11	NB-ARC domain-containing disease resistance protein	resistance protein LR10, putative, expressed
Bradi3g34697.5.p	NB-ARC,LRR	CNL;complete;17,16,1,6,4,5,10,3,12,2,8,7,9,11	NB-ARC domain-containing disease resistance protein	resistance protein LR10, putative, expressed
Bradi3g34697.6.p	NB-ARC,LRR	CNL;complete;17,16,1,6,4,5,10,3,12,2,8,7,9,11	NB-ARC domain-containing disease resistance protein	resistance protein LR10, putative, expressed
Bradi3g34697.7.p	NB-ARC,LRR	CNL;complete;17,16,1,6,4,5,10,3,12,2,8,7,9,11	NB-ARC domain-containing disease resistance protein	resistance protein LR10, putative, expressed
Bradi3g34697.8.p	NB-ARC,LRR	CNL;partial;4,5,10,3,12,2,8,7,9,11	NB-ARC domain-containing disease resistance protein	NBS-LRR disease resistance protein, putative, expressed
Bradi3g34722.1.p	NB-ARC,LRR	CNL;partial;17,16,1,6,4,5,10,3,12,2,8,7,9,9	NB-ARC domain-containing disease resistance protein	resistance protein LR10, putative, expressed
Bradi3g34723.1.p	NB-ARC,LRR	CNL;partial;1,6,4,5,10,3,12,2,8,7,11,9,9	NB-ARC domain-containing disease resistance protein	resistance protein, putative, expressed
Bradi3g34961.1.p	NB-ARC	CNL;partial;16,1,6,4,5,3,2,8,7,9	Concanavalin A-like lectin protein kinase family protein	RGH2B, putative, expressed
Bradi3g34961.2.p	NB-ARC	CNL;partial;16,1,6,4,5,3,2,8,7,9	NB-ARC domain-containing disease resistance protein	RGH2B, putative, expressed
Bradi3g34961.3.p	NB-ARC	CNL;partial;16,1,6,4,5,3,2,8,7,9	NB-ARC domain-containing disease resistance protein	RGH2B, putative, expressed
Bradi3g34967.1.p	NB-ARC	CNL;complete;17,16,1,4,3,2,8,11,9	NB-ARC domain-containing disease resistance protein	NB-ARC domain containing protein, expressed
Bradi3g34967.2.p	NB-ARC	CNL;complete;17,16,1,4,3,2,8,11,9	NB-ARC domain-containing disease resistance protein	NB-ARC domain containing protein, expressed
Bradi3g34967.3.p	NB-ARC	CNL;complete;17,16,1,4,3,2,8,11,9	NB-ARC domain-containing disease resistance protein	NB-ARC domain containing protein, expressed
Bradi3g38131.1.p	Not Found	Not Found	Ribonuclease H-like superfamily protein	NBS-LRR disease resistance protein, putative, expressed
Bradi3g39933.1.p	NB-ARC,LRR	CNL;partial;17,16,14,6,4,5,10,12,2,7,9,9,9,9,11	Disease resistance protein (CC-NBS-LRR class) family	RGH1A, putative, expressed
Bradi3g41810.1.p	LRR	N/A;partial;9,11,11,9,11,11,11,11,11,12	disease resistance family protein / LRR family protein	leucine rich repeat protein, putative, expressed
Bradi3g41865.1.p	NB-ARC	CNL;partial;17,16,1,6,4,15,3	NB-ARC domain-containing disease resistance protein	disease resistance protein RGA2, putative, expressed
Bradi3g41871.1.p	NB-ARC	Not Found	NB-ARC domain-containing disease resistance protein	disease resistance protein RGA1, putative, expressed
Bradi3g41876.1.p	LRR	N/A;partial;8,7,9,11,11,11,11,11,11,11,11,11,11	LRR and NB-ARC domains-containing disease resistance protein	CC-NBS-LRR, putative, expressed
Bradi3g41910.1.p	NB-ARC,LRR	CNL;complete;17,16,1,6,4,5,10,3,12,2,8,7,9,11,11,11,11,11,11,11,11,11,11	LRR and NB-ARC domains-containing disease resistance protein	disease resistance protein RGA2, putative, expressed
Bradi3g41960.1.p	NB-ARC,LRR	CNL;complete;17,16,1,6,4,5,10,3,12,2,8,7,9,11,11,11,11,11,11,11,11,11,11	LRR and NB-ARC domains-containing disease resistance protein	disease resistance protein RGA2, putative, expressed
Bradi3g42037.2.p	NB-ARC	CNL;partial;1,6,4,5,10,3,12,2,8,7,9	NB-ARC domain-containing disease resistance protein	MLA6 protein, putative, expressed
Bradi3g42037.3.p	NB-ARC	CNL;partial;1,6,4,5,10,3,12,2,8,7,9	NB-ARC domain-containing disease resistance protein	MLA6 protein, putative, expressed
Bradi3g42037.4.p	NB-ARC	CNL;partial;12,2,8,7,9	NB-ARC domain-containing disease resistance protein	MLA6 protein, putative, expressed
Bradi3g43577.3.p	Not Found	N/A;partial;15,13	Tetratricopeptide repeat (TPR)-like superfamily protein	tetratricopeptide repeat domain containing protein, expressed
Bradi3g44695.1.p	Not Found	Not Found	Ribonuclease H-like superfamily protein	NBS-LRR disease resistance protein, putative, expressed
Bradi3g45106.1.p	Not Found	Not Found	Polynucleotidyl transferase, ribonuclease H-like superfamily protein	NBS-LRR disease resistance protein, putative, expressed
Bradi3g45167.1.p	NB-ARC	CNL;complete;16,14,1,6,4,5,3,12,2,8,7,9,19,11	HOPZ-ACTIVATED RESISTANCE 1	NBS-LRR disease resistance protein, putative, expressed
Bradi3g45550.1.p	NB-ARC	Not Found	NB-ARC domain-containing disease resistance protein	expressed protein
Bradi3g49370.1.p	LRR	N/A;partial;11,11,11,11,9,11,11,11	phytosulfokin receptor 1	phytosulfokine receptor precursor, putative, expressed
Bradi3g49687.2.p	LRR	N/A;partial;9,11,11	RNI-like superfamily protein	F-box/LRR-repeat protein 2, putative, expressed
Bradi3g51110.1.p	LRR	N/A;partial;9,9,11,11,11,11,11,11,11,9,11,11,11	disease resistance family protein / LRR family protein	expressed protein
Bradi3g52220.1.p	Not Found	N/A;partial;13,1,1	P-glycoprotein 2	multidrug resistance protein, putative, expressed
Bradi3g52220.2.p	Not Found	N/A;partial;13,1,1	P-glycoprotein 2	multidrug resistance protein, putative, expressed
Bradi3g53350.1.p	LRR	N/A;partial;9,11,11,5	transmembrane kinase 1	receptor protein kinase TMK1 precursor, putative, expressed
Bradi3g55006.1.p	NB-ARC	CNL;complete;17,16,1,6,4,5,10,3,12,2,8,7,11	NB-ARC domain-containing disease resistance protein	NBS-LRR disease resistance protein, putative, expressed
Bradi3g56757.1.p	LRR	N/A;partial;9,9,9,9,11,9,9,9	Leucine-rich repeat (LRR) family protein	leucine-rich repeat-containing protein 40, putative, expressed
Bradi3g58937.5.p	NB-ARC	CNL;complete;17,16,1,6,4,5,10,3,12,2,8,7,11,9,9	NB-ARC domain-containing disease resistance protein	MLA10, putative, expressed
Bradi3g58951.2.p	NB-ARC	CNL;partial;16,1,6,4,5,3,12,2,8,7,9,9	NB-ARC domain-containing disease resistance protein	NB-ARC domain containing protein, expressed
Bradi3g60250.2.p	NB-ARC,LRR	CNL;complete;17,16,4,5,1,4,3,2,8,11,11,9	Disease resistance protein (CC-NBS-LRR class) family	resistance protein, putative, expressed
Bradi3g60337.1.p	NB-ARC,LRR	CNL;complete;16,1,6,4,5,3,12,2,7,11,11,11,11,11,11,11	NB-ARC domain-containing disease resistance protein	xa1, putative, expressed
Bradi3g60446.1.p	NB-ARC,LRR	CNL;complete;17,16,4,5,10,3,12,2,8,7,11,9,9	NB-ARC domain-containing disease resistance protein	resistance protein LR10, putative, expressed
Bradi3g60446.2.p	NB-ARC,LRR	CNL;partial;4,5,10,3,12,2,8,7,11,9,9	NB-ARC domain-containing disease resistance protein	resistance protein LR10, putative, expressed
Bradi3g60453.1.p	NB-ARC	Not Found	NB-ARC domain-containing disease resistance protein	resistance protein, putative, expressed
Bradi3g60981.1.p	NB-ARC	CNL;partial;16,1,6,4,5,2,8,7,11,9,9,9	NB-ARC domain-containing disease resistance protein	stripe rust resistance protein Yr10, putative, expressed
Bradi3g61040.2.p	NB-ARC	CNL;partial;6,4,5,10,3,2,8,7,3,19,9,11	NB-ARC domain-containing disease resistance protein	go35 NBS-LRR, putative, expressed
Bradi3g61040.3.p	NB-ARC	CNL;partial;3,2,8,7,3,19,9,11	LRR and NB-ARC domains-containing disease resistance protein	go35 NBS-LRR, putative, expressed
Bradi4g00600.1.p	NB-ARC,LRR	CNL;partial;17,12,2,8,7,9	Disease resistance protein (CC-NBS-LRR class) family	Leucine Rich Repeat family protein, expressed
Bradi4g00610.1.p	NB-ARC,LRR	CNL;partial;17,12,2,9,12,2,8,7,9	HOPZ-ACTIVATED RESISTANCE 1	expressed protein
Bradi4g00620.1.p	NB-ARC,LRR	CNL;partial;17,12,2,8,9	HOPZ-ACTIVATED RESISTANCE 1	expressed protein
Bradi4g01113.1.p	NB-ARC	CNL;partial;1,6,4,5,10,3,12,2,8,7,9,9	NB-ARC domain-containing disease resistance protein	NB-ARC domain containing protein, expressed
Bradi4g01113.2.p	NB-ARC	CNL;partial;1,6,4,5,10,3,12,2,8,7,9,9	NB-ARC domain-containing disease resistance protein	NB-ARC domain containing protein, expressed
Bradi4g01117.2.p	NB-ARC	CNL;partial;1,6,4,5,3,2,8,7,11,9,9	NB-ARC domain-containing disease resistance protein	resistance protein LR10, putative, expressed

Bradi4g01117.3.p	NB-ARC	CNL;partial;1,6,4,5,3,2,8,7,11,9,9	NB-ARC domain-containing disease resistance protein	resistance protein LR10, putative, expressed
Bradi4g01687.2.p	NB-ARC,LRR	CNL;complete;17,16,14,1,6,4,5,10,3,12,2,8,7,11,9,9,1	NB-ARC domain-containing disease resistance protein	RGH1A, putative, expressed
Bradi4g02490.2.p	NB-ARC	Not Found	LRR and NB-ARC domains-containing disease resistance protein	resistance protein, putative, expressed
Bradi4g02500.1.p	NB-ARC	TNL;partial;5,1,4,1,4,5,10,8,9	NB-ARC domain-containing disease resistance protein	resistance protein, putative, expressed
Bradi4g02500.2.p	NB-ARC	TNL;partial;5,1,4,1,4,5,10,8,9	Disease resistance protein (CC-NBS-LRR class) family	resistance protein, putative, expressed
Bradi4g02520.2.p	NB-ARC,LRR	CNL;complete;17,16,5,1,6,4,3,2,11,9,9,17	Disease resistance protein (CC-NBS-LRR class) family	resistance protein, putative, expressed
Bradi4g02525.6.p	NB-ARC	CNL;partial;17,16,1,6	Disease resistance protein (CC-NBS-LRR class) family	resistance protein LR10, putative, expressed
Bradi4g02525.7.p	NB-ARC	CNL;partial;17,16,1,6	Disease resistance protein (CC-NBS-LRR class) family	resistance protein LR10, putative, expressed
Bradi4g02525.8.p	NB-ARC	CNL;partial;17,16,1,6	Disease resistance protein (CC-NBS-LRR class) family	resistance protein LR10, putative, expressed
Bradi4g02525.9.p	NB-ARC	CNL;partial;17,16,1,6	NB-ARC domain-containing disease resistance protein	resistance protein LR10, putative, expressed
Bradi4g02528.1.p	NB-ARC	CNL;complete;17,16,1,4,5,10,3,12,2,8,7,11,11,9	NB-ARC domain-containing disease resistance protein	resistance protein LR10, putative, expressed
Bradi4g02528.2.p	NB-ARC	CNL;partial;4,5,10,3,12,2,8,7,11,11,9	NB-ARC domain-containing disease resistance protein	resistance protein, putative, expressed
Bradi4g02528.3.p	NB-ARC	CNL;partial;4,5,10,3,12,2,8,7,11,11,9	NB-ARC domain-containing disease resistance protein	resistance protein, putative, expressed
Bradi4g02535.2.p	NB-ARC	CNL;partial;17,16,1,6,4,5,10,3,12,2,8,9	Disease resistance protein (CC-NBS-LRR class) family	resistance protein LR10, putative, expressed
Bradi4g02541.1.p	LRR	Not Found	Disease resistance protein (CC-NBS-LRR class) family	Leucine Rich Repeat family protein, expressed
Bradi4g02544.1.p	NB-ARC	CNL;partial;17,16,1,6,10,12,2,8,7,11,9	Disease resistance protein (CC-NBS-LRR class) family	resistance protein LR10, putative, expressed
Bradi4g02552.1.p	NB-ARC	CNL;partial;17,16,14,1,6,4,5,10,3,12,2,8,7	NB-ARC domain-containing disease resistance protein	resistance protein LR10, putative, expressed
Bradi4g02620.1.p	Not Found	N/A;partial;9,11	RNI-like superfamily protein	Leucine Rich Repeat family protein, expressed
Bradi4g02625.1.p	NB-ARC	CNL;complete;17,16,1,6,4,5,10,3,12,2,8,7,11,9	Disease resistance protein (CC-NBS-LRR class) family	resistance protein LR10, putative, expressed
Bradi4g02825.1.p	NB-ARC	CNL;partial;17,16,14,1,6,4,5,10,3,12,2,8,7	NB-ARC domain-containing disease resistance protein	disease resistance protein RPM1, putative, expressed
Bradi4g03005.1.p	NB-ARC,LRR	CNL;complete;17,16,1,6,4,5,10,3,12,2,8,7,11,9,9,17	HOPZ-ACTIVATED RESISTANCE 1	resistance protein LR10, putative, expressed
Bradi4g03230.1.p	NB-ARC,LRR	Not Found	ADR1-like 1	disease resistance protein, putative, expressed
Bradi4g04655.2.p	NB-ARC,LRR	CNL;partial;17,16,14,1,6,4,5,10,3,12,2,8,7,9,9	HOPZ-ACTIVATED RESISTANCE 1	resistance protein LR10, putative, expressed
Bradi4g04655.3.p	NB-ARC,LRR	CNL;partial;17,16,14,1,6,4,5,10,3,12,2,8,7,9,9	HOPZ-ACTIVATED RESISTANCE 1	resistance protein LR10, putative, expressed
Bradi4g04655.4.p	NB-ARC,LRR	CNL;partial;17,16,14,1,6,4,5,10,3,12,2,8,7,9,9	HOPZ-ACTIVATED RESISTANCE 1	resistance protein LR10, putative, expressed
Bradi4g04657.1.p	NB-ARC,LRR	CNL;partial;17,16,14,1,6,4,5,10,3,12,2,8,7,9,9,9	NB-ARC domain-containing disease resistance protein	resistance protein LR10, putative, expressed
Bradi4g04662.2.p	NB-ARC,LRR	CNL;complete;17,16,14,1,6,4,5,10,3,12,2,8,7,11,9,9,9	NB-ARC domain-containing disease resistance protein	RGH1A, putative, expressed
Bradi4g04662.3.p	NB-ARC,LRR	CNL;complete;17,16,14,1,6,4,5,10,3,12,2,8,7,11,9,9,9	NB-ARC domain-containing disease resistance protein	RGH1A, putative, expressed
Bradi4g05385.1.p	Not Found	Not Found	RNA-directed DNA polymerase (reverse transcriptase)-related family protein	NBS-LRR disease resistance protein, putative, expressed
Bradi4g05870.1.p	NB-ARC,LRR	CNL;complete;17,16,1,6,4,5,3,12,2,8,7,11,11,11,11,11,11,11,11,11,11,11,11	LRR and NB-ARC domains-containing disease resistance protein	powdery mildew resistance protein PM3b, putative, expressed
Bradi4g06460.1.p	NB-ARC,LRR	CNL;partial;16,1,6,4,5,10,12,2,8,7,9,19,11,11,9,11,11,11,11	NB-ARC domain-containing disease resistance protein	NB-ARC domain containing protein, expressed
Bradi4g06470.1.p	NB-ARC,LRR	CNL;partial;16,1,6,4,5,12,2,8,7,19,11,11,11,11,11	LRR and NB-ARC domains-containing disease resistance protein	NB-ARC domain containing protein, expressed
Bradi4g06611.1.p	NB-ARC	CNL;partial;16,1,4,5,10,3,12,2,8,7	NB-ARC domain-containing disease resistance protein	NBS-LRR type disease resistance protein, putative, expressed
Bradi4g06880.1.p	NB-ARC,LRR	CNL;partial;17,12,2,8,7,9	NB-ARC domain-containing disease resistance protein	expressed protein
Bradi4g06970.1.p	NB-ARC,LRR	CNL;complete;17,16,1,6,4,5,10,3,12,2,8,7,9,9,9,11	NB-ARC domain-containing disease resistance protein	disease resistance protein RPM1, putative, expressed
Bradi4g07017.1.p	NB-ARC	CNL;partial;17,1,6,4,5,10,3,12,2,8,7,11,9,9	NB-ARC domain-containing disease resistance protein	MLA10, putative, expressed
Bradi4g07027.1.p	NB-ARC	CNL;partial;16,1,4,5,10,3,2,8,7,9	NB-ARC domain-containing disease resistance protein	NB-ARC domain containing protein, expressed
Bradi4g07027.2.p	NB-ARC	CNL;partial;5,10,3,2,8,7,9	LRR and NB-ARC domains-containing disease resistance protein	NB-ARC domain containing protein, expressed
Bradi4g07027.3.p	NB-ARC	CNL;partial;5,10,3,2,8,7,9	LRR and NB-ARC domains-containing disease resistance protein	NB-ARC domain containing protein, expressed
Bradi4g07027.4.p	NB-ARC	CNL;partial;5,3,2,8,7,9	Disease resistance protein (CC-NBS-LRR class) family	NB-ARC domain containing protein, expressed
Bradi4g07027.5.p	NB-ARC	CNL;partial;5,3,2,8,7,9	Disease resistance protein (CC-NBS-LRR class) family	NB-ARC domain containing protein, expressed
Bradi4g07655.1.p	Not Found	Not Found	Ribonuclease H-like superfamily protein	NBS-LRR disease resistance protein, putative, expressed
Bradi4g07902.2.p	NB-ARC	Not Found	NB-ARC domain-containing disease resistance protein	NB-ARC domain containing disease resistance protein, putative, expressed
Bradi4g07906.1.p	NB-ARC	TNL;partial;1,4,5	NB-ARC domain-containing disease resistance protein	NB-ARC domain containing disease resistance protein, putative, expressed
Bradi4g07911.1.p	NB-ARC	Not Found	LRR and NB-ARC domains-containing disease resistance protein	NB-ARC domain containing disease resistance protein, putative, expressed
Bradi4g08290.2.p	LRR	N/A;partial;4,11,9,11,11,11,11,11	Disease resistance protein (CC-NBS-LRR class) family	Leucine Rich Repeat family protein, expressed
Bradi4g08712.1.p	LRR	N/A;partial;6,9,11,11,9,11,11,11,11,11	NB-ARC domain-containing disease resistance protein	Leucine Rich Repeat family protein, expressed
Bradi4g09247.1.p	NB-ARC,LRR	CNL;complete;17,16,1,6,4,5,10,3,12,2,8,7,9,9,11,11	NB-ARC domain-containing disease resistance protein	disease resistance protein RPM1, putative, expressed
Bradi4g09265.1.p	Not Found	CNL;partial;17,16	NB-ARC domain-containing disease resistance protein	pollen signalling protein with adenylyl cyclase activity, putative, expressed
Bradi4g09271.1.p	NB-ARC,LRR	CNL;partial;3,2,8,7,9,9,11,19,11,11	PIF / Ping-Pong family of plant transposases	pollen signalling protein with adenylyl cyclase activity, putative, expressed
Bradi4g09312.1.p	NB-ARC,LRR	CNL;complete;17,16,1,6,4,5,10,3,2,8,7,9,11,11,11,11,10,13	NB-ARC domain-containing disease resistance protein	pollen signalling protein with adenylyl cyclase activity, putative, expressed
Bradi4g09530.1.p	NB-ARC,LRR	CNL;complete;17,16,1,6,4,5,10,3,2,8,7,9,11,9,19,20,11,13	NB-ARC domain-containing disease resistance protein	pollen signalling protein with adenylyl cyclase activity, putative, expressed
Bradi4g09577.1.p	NB-ARC,LRR	CNL;complete;16,1,6,4,5,10,3,2,8,7,11,9,9	NB-ARC domain-containing disease resistance protein	stripe rust resistance protein Yr10, putative, expressed
Bradi4g09577.2.p	NB-ARC,LRR	CNL;complete;16,1,6,4,5,10,3,2,8,7,11,9,9	NB-ARC domain-containing disease resistance protein	stripe rust resistance protein Yr10, putative, expressed
Bradi4g09587.4.p	NB-ARC,LRR	CNL;partial;17,16,14,1,6,4,5,10,3,12,2,8,7,9,9	NB-ARC domain-containing disease resistance protein	stripe rust resistance protein Yr10, putative, expressed
Bradi4g09587.5.p	NB-ARC,LRR	CNL;partial;17,16,14,1,6,4,5,10,3,12,2,8,7,9,9	NB-ARC domain-containing disease resistance protein	stripe rust resistance protein Yr10, putative, expressed
Bradi4g09597.1.p	NB-ARC,LRR	CNL;partial;17,16,1,6,4,5,10,3,12,2,8,7,9,9	NB-ARC domain-containing disease resistance protein	NB-ARC domain containing protein, expressed
Bradi4g09597.2.p	NB-ARC,LRR	CNL;partial;17,16,1,6,4,5,10,3,12,2,8,7,9,9	NB-ARC domain-containing disease resistance protein	NB-ARC domain containing protein, expressed
Bradi4g09597.3.p	NB-ARC,LRR	CNL;partial;17,16,1,6,4,5,10,3,12,2,8,7,9,9	NB-ARC domain-containing disease resistance protein	NB-ARC domain containing protein, expressed
Bradi4g09621.1.p	NB-ARC,LRR	CNL;partial;5,10,3,2,8,7,9,11,19,11,11,11	NB-ARC domain-containing disease resistance protein	pollen signalling protein with adenylyl cyclase activity, putative, expressed
Bradi4g09631.1.p	NB-ARC,LRR	CNL;complete;17,16,1,6,4,5,10,3,12,2,8,7,9,9,11,9,19,11,11	NB-ARC domain-containing disease resistance protein	pollen signalling protein with adenylyl cyclase activity, putative, expressed
Bradi4g09640.2.p	LRR	Not Found	NB-ARC domain-containing disease resistance protein	NBS-LRR disease resistance protein, putative, expressed
Bradi4g09648.1.p	NB-ARC,LRR	CNL;partial;3,12,2,8,7,9,9,11,9,19,11,11	PIF / Ping-Pong family of plant transposases	pollen signalling protein with adenylyl cyclase activity, putative, expressed
Bradi4g09652.1.p	NB-ARC	CNL;partial;17,16,1,6,4,5,10	HOPZ-ACTIVATED RESISTANCE 1	pollen signalling protein with adenylyl cyclase activity, putative, expressed
Bradi4g09660.2.p	LRR	Not Found	NB-ARC domain-containing disease resistance protein	NBS-LRR disease resistance protein, putative, expressed
Bradi4g09782.1.p	NB-ARC	Not Found	NB-ARC domain-containing disease resistance protein	NBS-LRR type disease resistance protein, putative, expressed
Bradi4g09788.1.p	NB-ARC	CNL;partial;1,6,4,5,10,3,2,8,7,11,11	NB-ARC domain-containing disease resistance protein	RGH2B, putative, expressed
Bradi4g09794.1.p	NB-ARC	CNL;partial;1,6,4,5,10,3,12,2,8,7,9,11	NB-ARC domain-containing disease resistance protein	NBS-LRR disease resistance protein, putative, expressed
Bradi4g09866.1.p	Not Found	CNL;partial;17,16,14	NB-ARC domain-containing disease resistance protein	resistance protein LR10, putative, expressed
Bradi4g09886.1.p	NB-ARC	CNL;partial;17,16,1,6,4,5,10,3,12,2,8,7	WRKY DNA-binding protein 33	RGH2B, putative, expressed
Bradi4g09886.2.p	NB-ARC	CNL;partial;17,16,1,6,4,5,10,3,12,2,8,7	WRKY DNA-binding protein 33	RGH2B, putative, expressed
Bradi4g09942.1.p	Not Found	N/A;partial;19,9,11	NB-ARC domain-containing disease resistance protein	go35 NBS-LRR, putative, expressed
Bradi4g09944.1.p	NB-ARC	CNL;partial;6,4,5,10,3,2,7,19,9,11	LRR and NB-ARC domains-containing disease resistance protein	go35 NBS-LRR, putative, expressed
Bradi4g09957.1.p	NB-ARC	CNL;complete;16,1,6,4,5,10,3,2,8,7,3,19,9,11	NB-ARC domain-containing disease resistance protein	go35 NBS-LRR, putative, expressed
Bradi4g10017.1.p	NB-ARC,LRR	CNL;partial;1,6,4,5,3,12,2,8,7,9,9,11,9,19,11,11,11	NB-ARC domain-containing disease resistance protein	NBS-LRR disease resistance protein, putative, expressed
Bradi4g10030.2.p	NB-ARC,LRR	CNL;complete;17,16,1,6,4,5,3,12,2,8,7,9,9,11,9,19,11,11,11,11,11	NB-ARC domain-containing disease resistance protein	NBS-LRR disease resistance protein, putative, expressed
Bradi4g10030.3.p	NB-ARC,LRR	CNL;complete;17,16,1,6,4,5,3,12,2,8,7,9,9,11,9,19,11,11,11,11,11	NB-ARC domain-containing disease resistance protein	NBS-LRR disease resistance protein, putative, expressed
Bradi4g10040.1.p	NB-ARC,LRR	CNL;partial;17,1,6,4,5,3,12,2,8,7,9,9,11,9,19,11,11,11,11,11	NB-ARC domain-containing disease resistance protein	NBS-LRR disease resistance protein, putative, expressed
Bradi4g10045.1.p	NB-ARC,LRR	CNL;complete;17,16,1,6,4,5,3,12,2,8,7,9,9,11,9,19,11,11,9,11	NB-ARC domain-containing disease resistance protein	NBS-LRR disease resistance protein, putative, expressed
Bradi4g10045.2.p	NB-ARC,LRR	CNL;complete;17,16,1,6,4,5,3,12,2,8,7,9,9,11,9,19,11,11,9,11	NB-ARC domain-containing disease resistance protein	NBS-LRR disease resistance protein, putative, expressed
Bradi4g10050.2.p	NB-ARC,LRR	CNL;partial;17,1,6,4,5,3,12,2,8,7,19,11,11,11	LRR and NB-ARC domains-containing disease resistance protein	NB-ARC domain containing protein, expressed
Bradi4g10055.1.p	Not Found	Not Found	Disease resistance protein (CC-NBS-LRR class) family	disease resistance protein, putative, expressed
Bradi4g10060.1.p	NB-ARC,LRR	CNL;complete;17,16,1,6,4,5,3,12,2,8,7,9,9,11,9,19,11,11,11,11,11	NB-ARC domain-containing disease resistance protein	NBS-LRR disease resistance protein, putative, expressed
Bradi4g10141.1.p	Not Found	CNL;partial;17,16	NB-ARC domain-containing disease resistance protein	disease resistance protein, putative, expressed
Bradi4g10149.1.p	NB-ARC	CNL;partial;17,16,1,6	NB-ARC domain-containing disease resistance protein	disease resistance protein, putative, expressed
Bradi4g10153.1.p	NB-ARC	CNL;partial;17,16,1,6,4,5	HOPZ-ACTIVATED RESISTANCE 1	disease resistance protein, putative, expressed
Bradi4g10153.2.p	NB-ARC	CNL;partial;17,16,1,6,4,5	HOPZ-ACTIVATED RESISTANCE 1	disease resistance protein, putative, expressed
Bradi4g10171.3.p	NB-ARC,LRR	CNL;complete;17,16,1,6,4,5,3,12,2,8,7,9,9,11,9,19,11,11,11,11,11	NB-ARC domain-containing disease resistance protein	NBS-LRR disease resistance protein, putative, expressed
Bradi4g10180.3.p	NB-ARC,LRR	CNL;complete;17,16,1,6,4,5,3,12,2,8,7,11,9,9,11,9,19,11,11,11,11,11	NB-ARC domain-containing disease resistance protein	NBS-LRR disease resistance protein, putative, expressed
Bradi4g10190.1.p	NB-ARC,LRR	CNL;partial;17,1,6,4,5,3,12,2,8,7,9,9,11,9,19,11,11,11,11	NB-ARC domain-containing disease resistance protein	NBS-LRR disease resistance protein, putative, expressed
Bradi4g10197.1.p	NB-ARC,LRR	CNL;partial;6,4,5,3,2,8,7,11,9,9,11,11	HOPZ-ACTIVATED RESISTANCE 1	NBS-LRR disease resistance protein, putative, expressed
Bradi4g10207.2.p	NB-ARC,LRR	CNL;complete;17,16,1,6,4,5,3,12,2,8,7,9,9,11,9,19,11,11,11,11,11	NB-ARC domain-containing disease resistance protein	NBS-LRR disease resistance protein, putative, expressed
Bradi4g10220.2.p	NB-ARC,LRR	CNL;partial;17,1,6,4,5,3,12,2,7,9,9,11,9,19,11,11,11	NB-ARC domain-containing disease resistance protein	NBS-LRR disease resistance protein, putative, expressed
Bradi4g10450.1.p	NB-ARC	CNL;partial;17,16,1,6,4,5,10,3,12,2,8,7	cysteine-rich RLK (RECEPTOR-like protein kinase) 19	RGH2B, putative, expressed
Bradi4g10450.2.p	NB-ARC	CNL;partial;17,16,1,6,4,5,10,3,12,2,8,7	S-locus lectin protein kinase family protein	RGH2B, putative, expressed
Bradi4g10453.1.p	NB-ARC	CNL;partial;17,16,1,4,5,3,2,7,9	NB-ARC domain-containing disease resistance protein	NB-ARC domain containing protein, expressed
Bradi4g10641.1.p	NB-ARC	CNL;partial;17,16,14,1,6,4,5,10,3,12,2,8,7	NB-ARC domain-containing disease resistance protein	stripe rust resistance protein Yr10, putative, expressed
Bradi4g10672.1.p	NB-ARC	CNL;partial;17,16,1,5,3,2,9	NB-ARC domain-containing disease resistance protein	NB-ARC domain containing protein, expressed
Bradi4g10675.1.p	NB-ARC,LRR	CNL;complete;17,16,14,1,6,4,5,10,3,12,2,8,7,11,9,11,20	Disease resistance protein (CC-NBS-LRR class) family	stripe rust resistance protein Yr10, putative, expressed
Bradi4g10987.1.p	LRR	N/A;partial;11,9,11,11,11,11,11	Leucine-rich repeat protein kinase family protein	receptor-like protein kinase 2 precursor, putative, expressed
Bradi4g10998.1.p	LRR	N/A;partial;11,9,11,11,11,11,11,17,11,11,9,11,9,11	Leucine-rich repeat protein kinase family protein	receptor-like protein kinase 2 precursor, putative, expressed
Bradi4g10998.2.p	LRR	N/A;partial;11,9,11,11,11,11,11,17,11,11,9,11,9,11	Leucine-rich repeat protein kinase family protein	receptor-like protein kinase 2 precursor, putative, expressed
Bradi4g10998.3.p	LRR	N/A;partial;11,9,11,11,11,11,11,11,11,9,11,9,11	Leucine-rich repeat protein kinase family protein	receptor-like protein kinase 2 precursor, putative, expressed

Bradi4g11338.1.p	NB-ARC,LRR	Not Found	Disease resistance protein (CC-NBS-LRR class) family	NBS-LRR type disease resistance protein, putative, expressed
Bradi4g11651.1.p	LRR	N/A;partial;9,11,11	Leucine-rich repeat (LRR) family protein	leucine-rich repeat family protein, putative, expressed
Bradi4g11920.2.p	NB-ARC,LRR	CNL;partial;1,6,4,5,2,7,9,9,11,11,11,19,11	NB-ARC domain-containing disease resistance protein	Leucine Rich Repeat family protein, expressed
Bradi4g11930.1.p	LRR	CNL;partial;2,8,7,11,11	Leucine-rich repeat (LRR) family protein	Leucine Rich Repeat family protein, expressed
Bradi4g11930.2.p	LRR	CNL;partial;2,8,7,11,11	Leucine-rich repeat (LRR) family protein	Leucine Rich Repeat family protein, expressed
Bradi4g11940.2.p	NB-ARC,LRR	CNL;partial;1,2,9,11,11,11,19,11,11	LRR and NB-ARC domains-containing disease resistance protein	Leucine Rich Repeat family protein, expressed
Bradi4g12131.1.p	NB-ARC,LRR	CNL;partial;17,16,1,6,4,5,2,8,7,9,11,9,19,11,11,11	NB-ARC domain-containing disease resistance protein	NBS-LRR disease resistance protein, putative, expressed
Bradi4g12313.1.p	NB-ARC,LRR	CNL;complete;17,16,1,6,4,5,3,2,8,7,9,9,11,9,19,11,11	NB-ARC domain-containing disease resistance protein	pollen signalling protein with adenylyl cyclase activity, putative, expressed
Bradi4g12666.1.p	NB-ARC,LRR	CNL;partial;1,6,4,5,3,2,8,9,9	NB-ARC domain-containing disease resistance protein	mla1, putative, expressed
Bradi4g12678.1.p	NB-ARC,LRR	CNL;partial;1,4,5,10,3,2,8,7,9,9,7	NB-ARC domain-containing disease resistance protein	stripe rust resistance protein Yr10, putative, expressed
Bradi4g12684.1.p	NB-ARC	CNL;partial;17,16,1,6,4,5,10,3,12,2,8,7,9,9	NB-ARC domain-containing disease resistance protein	NB-ARC domain containing protein, expressed
Bradi4g12730.2.p	Not Found	Not Found	NB-ARC domain-containing disease resistance protein	NBS-LRR type disease resistance protein, putative, expressed
Bradi4g12732.1.p	NB-ARC	CNL;complete;16,1,6,4,5,10,3,12,2,8,7,9,11	NB-ARC domain-containing disease resistance protein	RGH2B, putative, expressed
Bradi4g12734.1.p	NB-ARC	CNL;partial;1,6,5,10,3,2,8,11,9,9	NB-ARC domain-containing disease resistance protein	NB-ARC domain containing protein, expressed
Bradi4g12737.1.p	NB-ARC,LRR	CNL;partial;17,1,6,4,5,10,3,2,8,11,9,9	NB-ARC domain-containing disease resistance protein	stripe rust resistance protein Yr10, putative, expressed
Bradi4g12737.2.p	NB-ARC,LRR	CNL;partial;5,10,3,2,8,11,9,9	NB-ARC domain-containing disease resistance protein	stripe rust resistance protein Yr10, putative, expressed
Bradi4g12737.3.p	NB-ARC,LRR	CNL;partial;5,10,3,2,8,11,9,9	NB-ARC domain-containing disease resistance protein	stripe rust resistance protein Yr10, putative, expressed
Bradi4g12770.2.p	NB-ARC,LRR	CNL;partial;17,16,1,6,4,5,12,2,8,7,9	NB-ARC domain-containing disease resistance protein	RGH2B, putative, expressed
Bradi4g12770.3.p	NB-ARC,LRR	CNL;partial;17,16,1,6,4,5,12,2,8,7,9	NB-ARC domain-containing disease resistance protein	RGH2B, putative, expressed
Bradi4g12770.4.p	NB-ARC,LRR	CNL;partial;17,16,1,6,4,5,12,2,8,7,9	NB-ARC domain-containing disease resistance protein	RGH2B, putative, expressed
Bradi4g12785.1.p	NB-ARC	CNL;partial;1,4,5,2,8	Disease resistance protein (CC-NBS-LRR class) family	MLA10, putative, expressed
Bradi4g12877.1.p	NB-ARC,LRR	CNL;complete;17,16,14,1,6,4,5,10,3,12,2,8,7,11,9,11,9,19	NB-ARC domain-containing disease resistance protein	RGH1A, putative, expressed
Bradi4g12893.1.p	NB-ARC	CNL;complete;17,16,1,4,5,10,3,2,8,7,11,9,9	NB-ARC domain-containing disease resistance protein	NB-ARC domain containing protein, expressed
Bradi4g12895.1.p	NB-ARC	CNL;partial;16,1,6,4,5,10,3,12,2,8,7	NB-ARC domain-containing disease resistance protein	RGH2B, putative, expressed
Bradi4g13470.1.p	NB-ARC,LRR	CNL;partial;17,12,2,8,7,9,11	NB-ARC domain-containing disease resistance protein	Leucine Rich Repeat family protein, expressed
Bradi4g13480.1.p	NB-ARC,LRR	CNL;partial;17,12,2,7,9,11,9	NB-ARC domain-containing disease resistance protein	Leucine Rich Repeat family protein, expressed
Bradi4g13521.1.p	NB-ARC	CNL;partial;12,2,7,9,11	NB-ARC domain-containing disease resistance protein	Leucine Rich Repeat family protein, expressed
Bradi4g13533.1.p	NB-ARC	Not Found	HOPZ-ACTIVATED RESISTANCE 1	Leucine Rich Repeat family protein, expressed
Bradi4g13540.2.p	NB-ARC,LRR	CNL;partial;17,12,2,7,9,11	HOPZ-ACTIVATED RESISTANCE 1	Leucine Rich Repeat family protein, expressed
Bradi4g13550.1.p	NB-ARC,LRR	CNL;partial;17,12,2,7,9,11	NB-ARC domain-containing disease resistance protein	expressed protein
Bradi4g13600.1.p	LRR	N/A;partial;9,9,9,9,11,11,11,11	disease resistance family protein / LRR family protein	leucine rich repeat protein, putative, expressed
Bradi4g13975.1.p	Not Found	CNL;partial;17,16,1,7,9	NB-ARC domain-containing disease resistance protein	NB-ARC domain containing protein, expressed
Bradi4g13987.1.p	NB-ARC	CNL;partial;1,6,4,5,10,3,12,2,8,7,11	HOPZ-ACTIVATED RESISTANCE 1	RGH2B, putative, expressed
Bradi4g14104.1.p	NB-ARC,LRR	Not Found	NB-ARC domain-containing disease resistance protein	NB-ARC domain containing protein, putative, expressed
Bradi4g14697.1.p	NB-ARC,LRR	CNL;partial;1,6,4,5,10,3,12,2,8,7,9,9,9,11,9,11	NB-ARC domain-containing disease resistance protein	disease resistance protein RPM1, putative, expressed
Bradi4g15060.1.p	NB-ARC,LRR	CNL;partial;14,14,1,6,4,5,10,3,12,2,8,9,9,9,9,9	Disease resistance protein (CC-NBS-LRR class) family	NB-ARC domain containing protein, putative, expressed
Bradi4g15060.2.p	NB-ARC,LRR	CNL;partial;14,14,1,6,4,5,10,3,12,2,8,9,9,9,9,9	Disease resistance protein (CC-NBS-LRR class) family	NB-ARC domain containing protein, putative, expressed
Bradi4g15067.1.p	NB-ARC,LRR	CNL;complete;17,16,1,6,4,5,3,12,2,8,7,11,9,9	NB-ARC domain-containing disease resistance protein	LZ-NBS-LRR class, putative, expressed
Bradi4g16068.1.p	LRR	N/A;partial;11,11,11,9,11,9,11	Leucine-rich repeat protein kinase family protein	receptor kinase, putative, expressed
Bradi4g16130.2.p	LRR	N/A;partial;11,11,9,11,11	Leucine-rich repeat protein kinase family protein	receptor-like protein kinase 2 precursor, putative, expressed
Bradi4g16185.1.p	LRR	N/A;partial;9,9,9,9,11,11,11,11,11,11,11	disease resistance family protein / LRR family protein	leucine-rich repeat receptor protein kinase EXS precursor, putative, expressed
Bradi4g16242.1.p	LRR	N/A;partial;9,11,9,11,11,11,11,11,11,12	Leucine-rich receptor-like protein kinase family protein	Cf2/Cf5 disease resistance protein, putative, expressed
Bradi4g16340.1.p	NB-ARC	Not Found	Disease resistance protein (CC-NBS-LRR class) family	expressed protein
Bradi4g16492.2.p	NB-ARC,LRR	CNL;complete;17,16,1,6,4,5,10,3,12,2,8,7,11,9,9	Disease resistance protein (CC-NBS-LRR class) family	stripe rust resistance protein Yr10, putative, expressed
Bradi4g16492.3.p	NB-ARC,LRR	CNL;complete;17,16,1,6,4,5,10,3,12,2,8,7,11,9,9	Disease resistance protein (CC-NBS-LRR class) family	stripe rust resistance protein Yr10, putative, expressed
Bradi4g16492.4.p	NB-ARC,LRR	CNL;complete;17,16,1,6,4,5,10,3,12,2,8,7,11,9,9	Disease resistance protein (CC-NBS-LRR class) family	stripe rust resistance protein Yr10, putative, expressed
Bradi4g16492.5.p	NB-ARC,LRR	CNL;complete;17,16,1,6,4,5,10,3,12,2,8,7,11,9,9	Disease resistance protein (CC-NBS-LRR class) family	stripe rust resistance protein Yr10, putative, expressed
Bradi4g17141.1.p	NB-ARC,LRR	CNL;complete;17,16,1,6,4,5,3,2,8,7,9,11,19,11,11,11,11	NB-ARC domain-containing disease resistance protein	pollen signalling protein with adenylyl cyclase activity, putative, expressed
Bradi4g17365.1.p	NB-ARC,LRR	CNL;partial;17,1,6,4,5,10,3,12,2,8,7,9,9,11,11,11	NB-ARC domain-containing disease resistance protein	resistance protein, putative, expressed
Bradi4g20527.2.p	NB-ARC	CNL;complete;17,16,14,1,6,4,5,10,3,12,2,8,7,11,9	NB-ARC domain-containing disease resistance protein	MLA10, putative, expressed
Bradi4g20527.3.p	NB-ARC	CNL;complete;17,16,14,1,6,4,5,10,3,12,2,8,7,11,9	NB-ARC domain-containing disease resistance protein	MLA10, putative, expressed
Bradi4g21190.2.p	LRR	N/A;partial;9,11,11,11,11,11,9,11,11,11	Leucine-rich repeat transmembrane protein kinase	verticillium wilt disease resistance protein Ve2, putative, expressed
Bradi4g21190.3.p	LRR	N/A;partial;9,11,11,11,11,11,9,11,11,11	Leucine-rich repeat transmembrane protein kinase	verticillium wilt disease resistance protein Ve2, putative, expressed
Bradi4g21842.1.p	NB-ARC,LRR	CNL;complete;16,1,6,4,5,10,3,12,2,8,7,9,9,9,11	NB-ARC domain-containing disease resistance protein	disease resistance protein RPM1, putative, expressed
Bradi4g21890.1.p	NB-ARC,LRR	CNL;complete;16,1,6,4,5,10,3,12,2,8,7,9,9,9,9,11,11	NB-ARC domain-containing disease resistance protein	NB-ARC domain containing protein, expressed
Bradi4g21941.1.p	NB-ARC,LRR	Not Found	Disease resistance protein (CC-NBS-LRR class) family	resistance protein, putative, expressed
Bradi4g21950.2.p	NB-ARC	CNL;complete;17,16,6,4,5,1,6,4,5,3,2,8,11,9	Disease resistance protein (CC-NBS-LRR class) family	resistance protein, putative, expressed
Bradi4g21950.3.p	NB-ARC	CNL;complete;17,16,6,4,5,1,6,4,5,3,2,8,11,9	Disease resistance protein (CC-NBS-LRR class) family	resistance protein, putative, expressed
Bradi4g21950.4.p	NB-ARC	CNL;complete;17,16,6,4,5,1,6,4,5,3,2,8,11,9	Disease resistance protein (CC-NBS-LRR class) family	resistance protein, putative, expressed
Bradi4g21950.5.p	NB-ARC	CNL;complete;17,16,6,4,5,1,6,4,5,3,2,8,11,9	Disease resistance protein (CC-NBS-LRR class) family	resistance protein, putative, expressed
Bradi4g22014.1.p	NB-ARC	CNL;partial;17,16,1,4,5,10,3,12,2	NB-ARC domain-containing disease resistance protein	MLA10, putative, expressed
Bradi4g22740.1.p	NB-ARC	Not Found	NB-ARC domain-containing disease resistance protein	powdery mildew resistance protein PM3A, putative, expressed
Bradi4g23880.1.p	NB-ARC,LRR	CNL;complete;17,16,6,4,5,1,6,4,5,3,2,8,11,9	NB-ARC domain-containing disease resistance protein	resistance protein, putative, expressed
Bradi4g24461.1.p	LRR	N/A;partial;9,11,11	Leucine-rich repeat protein kinase family protein	expressed protein
Bradi4g24845.2.p	NB-ARC	Not Found	LRR and NB-ARC domains-containing disease resistance protein	expressed protein
Bradi4g24852.1.p	NB-ARC	Not Found	LRR and NB-ARC domains-containing disease resistance protein	expressed protein
Bradi4g24857.1.p	NB-ARC	Not Found	LRR and NB-ARC domains-containing disease resistance protein	expressed protein
Bradi4g24862.1.p	NB-ARC	Not Found	LRR and NB-ARC domains-containing disease resistance protein	expressed protein
Bradi4g24887.1.p	NB-ARC	CNL;complete;17,16,1,6,4,5,10,3,11,2,8,7,11,9	NB-ARC domain-containing disease resistance protein	NBS-LRR disease resistance protein, putative, expressed
Bradi4g24914.1.p	NB-ARC	CNL;complete;16,1,6,4,5,10,3,12,2,8,7,9,11	mitogen-activated protein kinase 1	NBS-LRR disease resistance protein, putative, expressed
Bradi4g24914.2.p	NB-ARC	CNL;partial;10,3,12,2,8,7,9,11	mitogen-activated protein kinase 1	CGMC_MAPKCMGC_2_SLT2y_ERK.2 - CGMC includes CDA, MAPK, GSK3, and CLKC kinases, expressed
Bradi4g24930.2.p	NB-ARC	Not Found	NB-ARC domain-containing disease resistance protein	NB-ARC domain containing disease resistance protein, putative, expressed
Bradi4g24959.1.p	Not Found	N/A;partial;9,11	F-box family protein	OsFBD10 - F-box and FBD domain containing protein, expressed
Bradi4g25034.1.p	NB-ARC,LRR	CNL;partial;5,10,3,12,2,8,7,11,11,9,11,11,9	Disease resistance protein (CC-NBS-LRR class) family	NB-ARC domain containing protein, expressed
Bradi4g25780.2.p	NB-ARC,LRR	CNL;partial;17,16,1,10,3,12,2,8,7,9,9,9	NB-ARC domain-containing disease resistance protein	NB-ARC domain containing protein, expressed
Bradi4g25810.1.p	NB-ARC,LRR	CNL;partial;17,16,1,10,3,12,2,8,7,9,9,9	NB-ARC domain-containing disease resistance protein	NB-ARC domain containing protein, expressed
Bradi4g28177.1.p	NB-ARC,LRR	CNL;complete;17,16,1,4,5,10,3,2,8,9,9,9,11,11,11,11,11,11	LRR and NB-ARC domains-containing disease resistance protein	Leucine Rich Repeat family protein, expressed
Bradi4g28177.2.p	NB-ARC,LRR	CNL;complete;17,16,1,4,5,10,3,2,8,9,9,9,11,11,11,11,11,11	LRR and NB-ARC domains-containing disease resistance protein	Leucine Rich Repeat family protein, expressed
Bradi4g28177.3.p	NB-ARC,LRR	CNL;complete;17,16,1,4,5,10,3,2,8,9,9,9,11,11,11,11,11,11	LRR and NB-ARC domains-containing disease resistance protein	Leucine Rich Repeat family protein, expressed
Bradi4g28177.4.p	NB-ARC,LRR	CNL;complete;17,16,1,4,5,10,3,2,8,9,9,9,11,11,11,11,11,11	LRR and NB-ARC domains-containing disease resistance protein	Leucine Rich Repeat family protein, expressed
Bradi4g28188.1.p	NB-ARC,LRR	CNL;complete;16,1,6,4,5,10,3,2,7,9,9,9,11,11,11,11,11,11,11,9,19,11,11,11	Protein kinase superfamily protein	Leucine Rich Repeat family protein, expressed
Bradi4g28324.1.p	NB-ARC,LRR	CNL;partial;2,8,7,9,9,11,9,19,11,11,11	NB-ARC domain-containing disease resistance protein	pollen signalling protein with adenylyl cyclase activity, putative, expressed
Bradi4g32786.1.p	Not Found	Not Found		NBS-LRR disease resistance protein, putative, expressed
Bradi4g33350.1.p	NB-ARC	Not Found	P-loop containing nucleoside triphosphate hydrolases superfamily protein	AP005392-AK108636 - NBS/LRR genes that are S-rich,divergent TIR, divergent NBS, expressed
Bradi4g33467.1.p	NB-ARC	CNL;complete;17,16,1,6,4,5,10,3,12,2,8,7,9,11	Disease resistance protein (CC-NBS-LRR class) family	NBS-LRR disease resistance protein, putative, expressed
Bradi4g33467.2.p	NB-ARC	CNL;complete;17,16,1,6,4,5,10,3,12,2,8,7,9,11	Disease resistance protein (CC-NBS-LRR class) family	NBS-LRR disease resistance protein, putative, expressed
Bradi4g33790.1.p	LRR	N/A;partial;9,11,9	Leucine-rich repeat (LRR) family protein	polygalacturonase inhibitor 1 precursor, putative, expressed
Bradi4g35317.1.p	NB-ARC,LRR	CNL;complete;17,16,4,5,10,3,12,2,8,7,9,9,11,8	NB-ARC domain-containing disease resistance protein	resistance protein, putative, expressed
Bradi4g36976.1.p	NB-ARC	CNL;partial;17,1,4,5,3,2,8,7,11,9	NB-ARC domain-containing disease resistance protein	NB-ARC domain containing protein, expressed
Bradi4g38170.2.p	NB-ARC,LRR	CNL;partial;17,1,6,4,5,3,12,2,8,7,9,9,9,19,11,11	LRR and NB-ARC domains-containing disease resistance protein	NB-ARC domain containing protein, expressed
Bradi4g38671.1.p	NB-ARC,LRR	CNL;complete;17,16,6,4,5,10,3,12,8,7,9,11,11,19,11,11,11	NB-ARC domain-containing disease resistance protein	pollen signalling protein with adenylyl cyclase activity, putative, expressed
Bradi4g39287.2.p	Not Found	CNL;partial;17,17,2,8,7,11	NB-ARC domain-containing disease resistance protein	expressed protein
Bradi4g39287.3.p	Not Found	CNL;partial;17,17,2,8,7,11	NB-ARC domain-containing disease resistance protein	expressed protein
Bradi4g39317.2.p	NB-ARC,LRR	CNL;complete;17,16,6,4,5,1,6,4,3,2,8,11,9	NB-ARC domain-containing disease resistance protein	resistance protein, putative, expressed
Bradi4g39317.3.p	NB-ARC,LRR	CNL;complete;17,16,6,4,5,1,6,4,3,2,8,11,9	NB-ARC domain-containing disease resistance protein	resistance protein, putative, expressed
Bradi4g39317.4.p	NB-ARC,LRR	CNL;complete;17,16,6,4,5,1,6,4,3,2,8,11,9	NB-ARC domain-containing disease resistance protein	resistance protein, putative, expressed
Bradi4g39317.5.p	NB-ARC,LRR	CNL;complete;17,16,6,4,5,1,6,4,3,2,8,11,9	NB-ARC domain-containing disease resistance protein	resistance protein, putative, expressed
Bradi4g40010.1.p	NB-ARC	Not Found	LRR and NB-ARC domains-containing disease resistance protein	expressed protein
Bradi4g40010.2.p	NB-ARC	Not Found	NB-ARC domain-containing disease resistance protein	expressed protein
Bradi4g40482.1.p	NB-ARC,LRR	CNL;complete;17,16,14,1,6,4,5,10,3,12,2,8,7,11,9,9	NB-ARC domain-containing disease resistance protein	resistance protein LR10, putative, expressed
Bradi4g40583.1.p	NB-ARC,LRR	CNL;partial;17,16,1,6,4,5,10,3,12,2,8,7,9,9	NB-ARC domain-containing disease resistance protein	NBS-LRR disease resistance protein, putative, expressed
Bradi4g40583.2.p	NB-ARC,LRR	CNL;partial;17,16,1,6,4,5,10,3,12,2,8,7,9,9	NB-ARC domain-containing disease resistance protein	NBS-LRR disease resistance protein, putative, expressed
Bradi4g40605.1.p	NB-ARC	Not Found	NB-ARC domain-containing disease resistance protein	rp3 protein, putative, expressed

Bradi4g40727.1.p	NB-ARC,LRR	CNL;partial;17,1,4,5,3,12,2,8,9,9,11,11	NB-ARC domain-containing disease resistance protein	resistance protein, putative, expressed
Bradi4g41708.1.p	NB-ARC,LRR	CNL;complete;16,1,6,4,5,3,12,2,8,7,11,11,9,9	NB-ARC domain-containing disease resistance protein	disease resistance protein RPM1, putative, expressed
Bradi4g44217.3.p	NB-ARC,LRR	CNL;partial;17,16,14,1,6,4,5,3,12,2,8,7,9,9	NB-ARC domain-containing disease resistance protein	stripe rust resistance protein Yr10, putative, expressed
Bradi4g44217.4.p	NB-ARC,LRR	CNL;partial;17,16,14,1,6,4,5,3,12,2,8,7,9,9	NB-ARC domain-containing disease resistance protein	stripe rust resistance protein Yr10, putative, expressed
Bradi4g44217.5.p	NB-ARC,LRR	CNL;partial;17,16,14,1,6,4,5,3,12,2,8,7,9,9	NB-ARC domain-containing disease resistance protein	stripe rust resistance protein Yr10, putative, expressed
Bradi4g44227.1.p	NB-ARC,LRR	CNL;partial;17,16,14,1,6,4,5,10,3,12,2,8,7,9,9,9	NB-ARC domain-containing disease resistance protein	stripe rust resistance protein Yr10, putative, expressed
Bradi4g44227.2.p	NB-ARC,LRR	CNL;partial;17,16,14,1,6,4,5,10,3,12,2,8,7,9,9,9	NB-ARC domain-containing disease resistance protein	stripe rust resistance protein Yr10, putative, expressed
Bradi4g44227.3.p	NB-ARC,LRR	CNL;partial;17,16,14,1,6,4,5,10,3,12,2,8,7,9,9,9	NB-ARC domain-containing disease resistance protein	stripe rust resistance protein Yr10, putative, expressed
Bradi4g44227.4.p	NB-ARC,LRR	CNL;partial;17,16,14,1,6,4,5,10,3,12,2,8,7,9,9,9	NB-ARC domain-containing disease resistance protein	stripe rust resistance protein Yr10, putative, expressed
Bradi4g44550.1.p	NB-ARC,LRR	CNL;partial;17,16,14,1,6,1,6,4,5,10,3,12,2,8,7,9,9	HOPZ-ACTIVATED RESISTANCE 1	mla1, putative, expressed
Bradi4g44550.3.p	NB-ARC,LRR	CNL;partial;17,16,14,1,6,4,5,10,3,12,2,8,7,9,9	NB-ARC domain-containing disease resistance protein	stripe rust resistance protein Yr10, putative, expressed
Bradi4g44550.4.p	NB-ARC,LRR	CNL;partial;17,16,14,1,6,4,5,10,3,12,2,8,7,9,9	NB-ARC domain-containing disease resistance protein	stripe rust resistance protein Yr10, putative, expressed
Bradi4g44550.5.p	NB-ARC,LRR	CNL;partial;17,16,14,1,6,4,5,10,3,12,2,8,7,9,9	NB-ARC domain-containing disease resistance protein	stripe rust resistance protein Yr10, putative, expressed
Bradi4g44575.1.p	NB-ARC,LRR	CNL;partial;17,16,14,1,6,4,5,10,3,12,2,8,7,9,9	NB-ARC domain-containing disease resistance protein	stripe rust resistance protein Yr10, putative, expressed
Bradi4g44575.2.p	NB-ARC,LRR	CNL;partial;17,16,14,1,6,4,5,10,3,12,2,8,7,9,9	NB-ARC domain-containing disease resistance protein	stripe rust resistance protein Yr10, putative, expressed
Bradi4g44590.1.p	NB-ARC	CNL;partial;17,16,14,1,6	Disease resistance protein (CC-NBS-LRR class) family	RGH1A, putative, expressed
Bradi4g44590.2.p	NB-ARC	CNL;partial;17,16,14,1,6	Disease resistance protein (CC-NBS-LRR class) family	mla1, putative, expressed
Bradi4g44603.1.p	NB-ARC,LRR	CNL;partial;17,16,14,1,6,4,5,10,3,12,2,8,7,9,9	NB-ARC domain-containing disease resistance protein	stripe rust resistance protein Yr10, putative, expressed
Bradi4g44642.1.p	NB-ARC	CNL;partial;14,1,6,4,5,10,3,12,2	NB-ARC domain-containing disease resistance protein	RGH1A, putative, expressed
Bradi4g44646.1.p	NB-ARC	CNL;partial;17,16,14,1	NB-ARC domain-containing disease resistance protein	mla1, putative, expressed
Bradi5g00451.1.p	NB-ARC	CNL;complete;17,16,1,6,4,3,12,8,7,11,19,11,19,11,11,11	LRR and NB-ARC domains-containing disease resistance protein	powdery mildew resistance protein PM3b, putative, expressed
Bradi5g00616.1.p	NB-ARC,LRR	N/A;partial;11,11,9,11,11,11,11	Disease resistance protein (TIR-NBS-LRR class) family	Leucine Rich Repeat family protein, expressed
Bradi5g00616.2.p	NB-ARC,LRR	N/A;partial;11,11,9,11,11,11,11	Disease resistance protein (TIR-NBS-LRR class) family	Leucine Rich Repeat family protein, expressed
Bradi5g00622.1.p	LRR	N/A;partial;11,11,9,11,11,11,11	NB-ARC domain-containing disease resistance protein	Leucine Rich Repeat family protein, expressed
Bradi5g00871.1.p	NB-ARC	CNL;complete;16,1,6,4,5,10,3,12,2,8,7,9,19,11,11,11,11,11,11	LRR and NB-ARC domains-containing disease resistance protein	NBS-LRR disease resistance protein, putative, expressed
Bradi5g01070.1.p	NB-ARC	Not Found	NB-ARC domain-containing disease resistance protein	expressed protein
Bradi5g01080.2.p	NB-ARC	Not Found		expressed protein
Bradi5g01101.1.p	LRR	N/A;partial;11,11,11,11,11,11,9,11,11	disease resistance protein (TIR-NBS-LRR class)	rp3 protein, putative, expressed
Bradi5g01167.1.p	NB-ARC	CNL;complete;16,1,6,4,5,10,3,12,2,8,7,9,9,9,9,11,11	NB-ARC domain-containing disease resistance protein	disease resistance protein RPM1, putative, expressed
Bradi5g01205.1.p	NB-ARC,LRR	CNL;partial;1,6,5,10,3,12,7,9,9,11,11,11,11	NB-ARC domain-containing disease resistance protein	disease resistance protein RPS2, putative, expressed
Bradi5g01210.1.p	LRR	N/A;partial;11,11,11,9,11,11,11,11,11,6	Leucine-rich repeat receptor-like protein kinase family protein	receptor-like protein kinase 2 precursor, putative, expressed
Bradi5g01457.1.p	NB-ARC	Not Found		NB-ARC domain containing protein, expressed
Bradi5g01470.2.p	NB-ARC	Not Found	NB-ARC domain-containing disease resistance protein	NB-ARC domain containing protein, expressed
Bradi5g01480.1.p	NB-ARC	Not Found	NB-ARC domain-containing disease resistance protein	NB-ARC domain containing protein, expressed
Bradi5g01546.1.p	NB-ARC,LRR	CNL;complete;17,16,6,4,5,1,4,5,10,3,2,8,11,9	NB-ARC domain-containing disease resistance protein	resistance protein, putative, expressed
Bradi5g01923.1.p	Not Found	Not Found		CC-NBS-LRR protein, putative, expressed
Bradi5g01936.1.p	NB-ARC,LRR	CNL;partial;1,6,4,5,10,3,12,2,8,7,9,11,11,11,11,11,11	LRR and NB-ARC domains-containing disease resistance protein	disease resistance protein RGA3, putative, expressed
Bradi5g02240.2.p	LRR	N/A;partial;11,9,11,11,11,11,11,11	disease resistance protein (TIR-NBS-LRR class), putative	Leucine Rich Repeat family protein, expressed
Bradi5g02360.1.p	NB-ARC	CNL;partial;17,1,6,4,5,3,12,2,8,7,9,11,11	NB-ARC domain-containing disease resistance protein	NBS-LRR disease resistance protein, putative, expressed
Bradi5g02360.2.p	NB-ARC	CNL;partial;17,1,6,4,5,3,12,2,8,7,9,11,11	NB-ARC domain-containing disease resistance protein	NBS-LRR disease resistance protein, putative, expressed
Bradi5g02367.1.p	NB-ARC,LRR	CNL;partial;1,6,4,5,10,3,12,2,8,7,11,11,11,11,11,11,11	NB-ARC domain-containing disease resistance protein	NB-ARC domain containing protein, expressed
Bradi5g02426.1.p	Not Found	Not Found	Ribonuclease H-like superfamily protein	NBS-LRR disease resistance protein, putative, expressed
Bradi5g02430.1.p	LRR	N/A;partial;9,11,11,11,11,11	Disease resistance protein (CC-NBS-LRR class) family	Leucine Rich Repeat family protein, expressed
Bradi5g02453.1.p	Not Found	Not Found	Ribonuclease H-like superfamily protein	NBS-LRR disease resistance protein, putative, expressed
Bradi5g02860.2.p	NB-ARC,LRR	CNL;complete;16,1,6,4,5,10,3,12,2,8,7,9,11,19,11,11,11	NB-ARC domain-containing disease resistance protein	disease resistance RPP13-like protein 1, putative, expressed
Bradi5g03110.1.p	NB-ARC,LRR	CNL;complete;16,1,6,4,5,10,3,12,2,8,7,9,9,9,11,11	NB-ARC domain-containing disease resistance protein	disease resistance protein RPM1, putative, expressed
Bradi5g03140.1.p	NB-ARC,LRR	CNL;partial;16,1,6,4,5,10,2,8,7,9,9,9,11	NB-ARC domain-containing disease resistance protein	NB-ARC domain containing protein, expressed
Bradi5g03559.1.p	Not Found	Not Found	RNA-directed DNA polymerase (reverse transcriptase)-related family protein	NBS-LRR disease resistance protein, putative, expressed
Bradi5g03986.1.p	NB-ARC	CNL;partial;17,16,14,1,3,2	Disease resistance protein (CC-NBS-LRR class) family	resistance protein, putative, expressed
Bradi5g03989.1.p	NB-ARC,LRR	CNL;complete;17,16,14,1,6,4,5,10,3,12,2,8,7,11,9,9	NB-ARC domain-containing disease resistance protein	resistance protein LR10, putative, expressed
Bradi5g10277.2.p	LRR	N/A;partial;9,11,11,11,9,11,11,11,11,11	NB-ARC domain-containing disease resistance protein	Leucine Rich Repeat family protein, expressed
Bradi5g10531.1.p	Not Found	Not Found	RNA-directed DNA polymerase (reverse transcriptase)-related family protein	NBS-LRR disease resistance protein, putative, expressed
Bradi5g13170.1.p	LRR	N/A;partial;9,11,11	Leucine-rich repeat receptor-like protein kinase family protein	receptor protein kinase CLAVATA1 precursor, putative, expressed
Bradi5g13170.2.p	LRR	N/A;partial;9,11,11	Leucine-rich repeat receptor-like protein kinase family protein	receptor protein kinase CLAVATA1 precursor, putative, expressed
Bradi5g13617.1.p	Not Found	N/A;partial;1,13,1	ABC transporter family protein	ABC transporter, ATP-binding protein, putative, expressed
Bradi5g15027.2.p	LRR	N/A;partial;11,11,11,9,11,11,11,11,11	Leucine-rich repeat protein kinase family protein	receptor-like protein kinase precursor, putative, expressed
Bradi5g15027.3.p	LRR	N/A;partial;11,11,11,9,11,11,11,11,11	Leucine-rich repeat protein kinase family protein	receptor-like protein kinase precursor, putative, expressed
Bradi5g15560.1.p	NB-ARC,LRR	CNL;partial;1,4,5,10,3,12,2,7,11,9,9,11,11,11,11	NB-ARC domain-containing disease resistance protein	NB-ARC/LRR disease resistance protein, putative, expressed
Bradi5g15565.1.p	NB-ARC,LRR	CNL;partial;1,4,5,10,3,12,2,7,11,9,9,11,11,11,11,11,11	Disease resistance protein (CC-NBS-LRR class) family	NB-ARC/LRR disease resistance protein, putative, expressed
Bradi5g17527.1.p	NB-ARC,LRR	CNL;partial;1,6,4,5,10,3,12,2,8,7,9,9,9,9,11,11,11	NB-ARC domain-containing disease resistance protein	disease resistance protein RPM1, putative, expressed
Bradi5g21870.1.p	LRR	N/A;partial;11,11,11,9,11,12	Leucine-rich repeat transmembrane protein kinase	SHR5-receptor-like kinase, putative, expressed
Bradi5g21870.2.p	LRR	N/A;partial;11,11,11,9,11,12	Leucine-rich repeat transmembrane protein kinase	SHR5-receptor-like kinase, putative, expressed
Bradi5g22146.1.p	NB-ARC,LRR	CNL;complete;16,1,6,4,5,10,3,12,2,8,7,19,11,11,9,11,11,11,11,11	NB-ARC domain-containing disease resistance protein	NBS-LRR disease resistance protein, putative, expressed
Bradi5g22146.2.p	NB-ARC,LRR	CNL;complete;16,1,6,4,5,10,3,12,2,8,7,19,11,11,9,11,11,11,11,11	NB-ARC domain-containing disease resistance protein	NBS-LRR disease resistance protein, putative, expressed
Bradi5g22162.1.p	NB-ARC,LRR	CNL;complete;16,1,6,4,5,10,3,12,2,8,19,11,11,11,11,11	NB-ARC domain-containing disease resistance protein	NBS-LRR disease resistance protein, putative, expressed
Bradi5g22176.1.p	NB-ARC	CNL;partial;16,1,6,4,5,10,3,12	NB-ARC domain-containing disease resistance protein	NBS-LRR disease resistance protein, putative, expressed
Bradi5g22178.1.p	LRR	Not Found	NB-ARC domain-containing disease resistance protein	NBS-LRR disease resistance protein, putative, expressed
Bradi5g22187.1.p	NB-ARC,LRR	CNL;complete;16,1,6,4,5,10,3,12,2,8,7,9,11,11,11,11,11,11,11,11,11,11,11,11,11,11	NB-ARC domain-containing disease resistance protein	NBS-LRR disease resistance protein, putative, expressed
Bradi5g22547.1.p	NB-ARC	CNL;complete;16,1,6,4,5,3,12,2,8,7,16,19,11,11,11,11,11,11,11,11	LRR and NB-ARC domains-containing disease resistance protein	NBS-LRR disease resistance protein, putative, expressed
Bradi5g22842.1.p	NB-ARC	CNL;complete;16,1,6,4,5,3,12,2,8,7,16,19,11,11,11,11,11,11,11,11	LRR and NB-ARC domains-containing disease resistance protein	NBS-LRR disease resistance protein, putative, expressed
Bradi5g22866.1.p	Not Found	Not Found	disease resistance protein (TIR-NBS-LRR class)	NBS-LRR disease resistance protein, putative, expressed
Bradi5g25030.1.p	LRR	N/A;partial;9,11,9,11,11,11,11	disease resistance family protein / LRR family protein	leucine rich repeat protein, putative, expressed
Bradi5g25790.1.p	LRR	N/A;partial;11,11,11,11,11,9,11,11,11	phytosulfokin receptor 1	phytosulfokine receptor precursor, putative, expressed
Bradi5g27069.1.p	NB-ARC,LRR	CNL;complete;17,16,14,1,6,4,5,10,3,12,2,8,7,9,9,11,17	NB-ARC domain-containing disease resistance protein	stripe rust resistance protein Yr10, putative, expressed
Bradi5g27426.1.p	Not Found	Not Found	Ribonuclease H-like superfamily protein	NBS-LRR disease resistance protein, putative, expressed
